# 85 °C/85%‐Stable n‐i‐p Perovskite Photovoltaics with NiO*_x_* Hole Transport Layers Promoted By Perovskite Quantum Dots

**DOI:** 10.1002/advs.202201573

**Published:** 2022-07-20

**Authors:** Fangwen Cheng, Fang Cao, Binwen Chen, Xinfeng Dai, Ziheng Tang, Yifei Sun, Jun Yin, Jing Li, Nanfeng Zheng, Binghui Wu

**Affiliations:** ^1^ State Key Laboratory for Physical Chemistry of Solid Surfaces Collaborative Innovation Center of Chemistry for Energy Materials (iChEM) National & Local Joint Engineering Research Center of Preparation Technology of Nanomaterials College of Chemistry and Chemical Engineering Pen‐Tung Sah Institute of Micro‐Nano Science and Technology College of Energy Jiujiang Research Institute Innovation Laboratory for Sciences and Technologies of Energy Materials of Fujian Province (IKKEM) Xiamen University Xiamen 361005 China

**Keywords:** interfacial engineering, NiO*_x_* hole transport layers, n‐i‐p perovskite solar cells, perovskite quantum dots

## Abstract

Power conversion efficiency (PCE) and long‐term stability are two vital issues for perovskite solar cells (PSCs). However, there is still a lack of suitable hole transport layers (HTLs) to endow PSCs with both high efficiency and stability. Here, NiO*_x_* nanoparticles are promoted as an efficient and 85 °C/85%‐stable inorganic HTL for high‐performance n‐i‐p PSCs, with the introduction of perovskite quantum dots (QDs) between perovskite and NiO*_x_* as systematic interfacial engineering. The QD intercalation enhances film morphology and assembly regulation of NiO*_x_* HTLs . Due to structure–function correlations, hole mobility within NiO*_x_* HTL is improved. And the hole extraction from perovskite to NiO*_x_* is also facilitated, resulting from reduced trap states and optimized energy level alignments. Hence, the promoted NiO*_x_*‐based n‐i‐p PSCs exhibit high PCE (21.59%) and excellent stability (sustaining 85 °C aging in air without encapsulation). Furthermore, encapsulated solar modules with QDs‐promoted NiO*_x_* HTLs show impressive stability during 85 °C/85% aging test for 1000 hours. With high transparency, QDs‐promoted NiO*_x_* is also demonstrated to be an advanced HTL for semitransparent PSCs. This work develops promising NiO*_x_* inorganic HTL in n‐i‐p PSCs for manufacturing next‐generation photovoltaic devices.

## Graphical abstract

The intercalation of perovskite quantum dots between perovskites and NiO*_x_* hole transport layers is introduced to promote steric arrangements and electronic properties of NiO*_x_* nanoparticles. The performances of NiO*_x_*‐based n‐i‐p perovskite solar devices are significantly improved, realizing high efficiency (21.59%) and excellent stability (sustaining 85 °C/85% RH aging test).

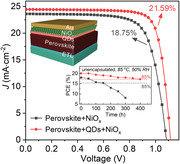

## 1 Introduction

Perovskite solar cells (PSCs) have reached remarkable certified power conversion efficiencies (PCEs) up to 25.7% with n‐i‐p configuration.^[^ ^1^ ^]^ In the n‐i‐p devices, p‐type organic semiconductors (e.g., spiro‐OMeTAD,^[^ ^2^ ^]^ PTAA,^[^ ^3^ ^]^ P3HT^[^ ^4^ ^]^) have been usually chosen as hole transport layers (HTLs) to achieve high PCEs. However, the intrinsic photothermal instability or external hydrophilic ionic doping of these organic HTLs limits their further application in commercial manufacture.^[^ ^5^ ^]^ To solve this stability issue, adopting inorganic HTLs (io‐HTLs) with great thermal stability has been considered as a promising solution.^[^ ^6^ ^]^ Up till now, most reports of io‐HTLs in n‐i‐p PSCs have focused on Cu‐based inorganic semiconductors.^[^ ^7^ ^]^ But the stability concerns still exist, due to the oxidation tendency of Cu(I) ions^[^ ^8^ ^]^ and the chemical reactivity between the anions (e.g., SCN^−^, I^−^) and metal electrodes.^[^ ^9^ ^]^ Furthermore, their deposition methods are not feasible for practical process, due to the inevitable toxic solvents^[^ ^10^ ^]^ (e.g., sulfide) or uncontrollable post treatments^[^ ^11^ ^]^ (e.g., gas–solid and solid–solid reactions). Therefore, finding a stable, processable and chemically compatible io‐HTL for n‐i‐p devices is of great significance.

As an inorganic oxide semiconductor, p‐type NiO*_x_* shows its advantages in efficient and stable p‐i‐n PSCs,^[^ ^12^ ^]^ benefitting from its high hole mobility,^[^ ^13^ ^]^ matched energy level with perovskite^[^ ^14^ ^]^ and good stability against moisture and heat.^[^ ^15^ ^]^ In most cases, dense NiO*_x_* HTLs on FTO/ITO were prepared by pyrolysis of sol–gel precursors over 300 °C,^[^ ^16^ ^]^ O_3_‐involved atomic layer deposition^[^ ^17^ ^]^ or deposition of pre‐synthesized aqueous NiO*_x_* nanoparticles (NPs).^[^ ^12^, ^18^ ^]^ However, these above processes are not applicable for the deposition of NiO*_x_* HTLs on the perovskites in n‐i‐p devices. Instead, NiO*_x_* NPs with oleophilic ligands that can be dissolved in nonpolar solvents have been achieved in previous work for the deposition of NiO*_x_* on perovskite layers. But the corresponding devices showed inferior efficiencies (<16%) and unsatisfactory stabilities, due to the incompact assembly and low hole transport capability of NiO*_x_* NPs on perovskites.^[^ ^19^ ^]^ Therefore, realizing uniform and ordered NiO*_x_* films on perovskite surface is a key point in fabricating high‐performance NiO*_x_*‐based n‐i‐p devices.

In this work, we aim to find an effective strategy to improve steric arrangement of NiO*_x_* NPs to form uniform and ordered NiO*_x_* HTLs on perovskites. As reported, the properties of the substrate's surface influenced the assembly behavior of nanoparticles.^[^ ^20^ ^]^ Therefore, we assumed that perovskite quantum dots (QDs) with long‐chain oleophilic would tune the surface properties of perovskite layer and thus regulate the assembly behavior of NiO*_x_* NPs. For the NiO*_x_* HTLs, QD intercalation improved the film formation and regulation of NiO*_x_* NPs, along with p‐type properties due to structure–function correlation. For the perovskite layers, QD intercalation passivated the trap states and optimized the energy alignments of perovskite and NiO*_x_*, thus accelerating the hole extaction and reducing nonradiative recombination. Through systematic interfacial engineering, the devices fabricated with QDs‐promoted NiO*_x_* HTLs exhibited high efficiencies and excellent stabilities. The superiority of QDs‐promoted NiO*_x_* HTLs was further extended to opaque large‐scale perovskite solar modules and small‐area semitransparent perovskite solar devices, exhibiting excellent performances (survived at 85 °C/85% relative humidity (RH) for 1000 h). To our best knowledge, this is the first case using NiO*_x_* HTLs in n‐i‐p type solar modules and semitransparent cells with remarkable performance. This work shows great potential of QDs‐promoted NiO*_x_* HTLs in future commercial application of perovskite photovoltaics.

## 2 Results and Discussions

### 2.1 Steric and Electronic Improvement of NiO*_x_* HTLs By QD Intercalation

**Figure 1:**
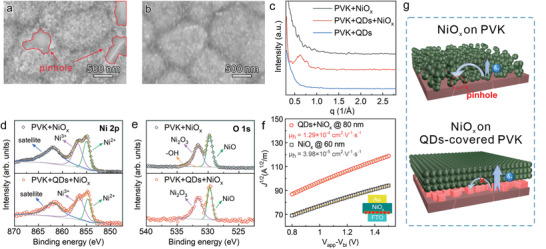
Quality improvement of NiO*_x_* HTLs with QDs on perovskites. a,b) SEM images of NiO*_x_* film on perovskite or QDs‐covered perovskite. c) SAXS spectra of the corresponding films. d,e) XPS of Ni 2p and O 1s of NiO*_x_* HTLs. f) Hole mobilities of NiO*_x_* HTLs. g) Statement schematics for steric configuration of NiO*_x_* HTLs deposited on perovskite or QDs‐covered perovskite.

After confirming the successful synthesis of oleophilic NiO*_x_* NPs (Figures S1–S3, Supporting Information) and CsPbI_1.85_Br_1.15_ QDs (Figure S4, Supporting Information), we deposited NiO*_x_* HTLs by spin‐coating NiO*_x_* NPs on perovskites or QDs‐covered perovskite films. As reported, the uniformity of NiO*_x_* films on perovskites is an important factor affecting device's performance.^[^ ^19a^ ^]^ However, the pristine NiO*_x_* film showed poor morphology when being deposited directly on perovskite. Most of the NiO*_x_* NPs aggregated at the grain boundaries of perovskite (**Figure** **1**a), leading to nonradiative charge recombination centers and instability concerns due to direct contact between perovskite and electrode. To overcome the problem, QD intercalation was introduced to assist NiO*_x_* film formation. NiO*_x_* NPs could form a uniform and compact film on QDs‐covered perovskite surface (Figure 1b). The QDs‐promoted NiO*_x_* film showed improved uniformity with decreased roughness, as attested by atomic force microscopy (AFM) and energy‐dispersive X‐ray spectroscopy (EDX) (Figures S5 and S6, Supporting Information). The homogeneous NiO*_x_* film avoided direct contact between perovskite and Au electrode in n‐i‐p devices (Figure S7, Supporting Information). Besides improved film coverage, NiO*_x_* NPs showed promoted self‐assembly behavior with QD intercalation. From small angle X‐ray scattering (SAXS) spectra of NiO*_x_* films (Figure 1c), a scattering peak raised at 0.6 1/Å for the QDs‐promoted NiO*_x_* film, indicating the enhanced regularity of NiO*_x_* domains; while no evident peak was shown for pristine NiO*_x_* film.^[^ ^21^ ^]^ The improvement of NiO*_x_* assembly behavior resulted from beneficial interaction between QDs and stearate ligands on NiO*_x_* nanoparticles (Figure S8, Supporting Information).

Due to optimized steric regulation of NiO*_x_* film and structure–function correlation, the electronic status of NiO*_x_* HTL was altered after QD promotion. As shown in X‐ray photoelectron spectroscopy (XPS) spectra, NiO*_x_* films were verified to be nonstoichiometric, consisting of Ni^2+^ and Ni^3+^ simultaneously (Figure 1d,e). Ni^3+^ ions were reported to result from holes around Ni^2+^ vacancies in the lattice and generate p‐type conductivity in the NiO*_x_* films.^[^ ^22^ ^]^ This means the higher concentration of Ni^3+^ in QDs‐promoted NiO*_x_* film is conducive to hole transport. Compared with the pristine NiO*_x_*, the QDs‐promoted NiO*_x_* exhibited a higher ratio of Ni^3+^/Ni^2+^ (raised from 1.38 to 1.96). QD intercalation influences binding states of stearate ligands on NiO_x_, altering electronic status of Ni^2+^/Ni^3+^ (Figure S8, Supporting Information). In addition, there was a noticeable reduction of hydroxyl groups in NiO*_x_* films with QD intercalation, which could be attributed to higher ligand coverage of NiO*_x_* NPs. The uniform QDs‐promoted NiO*_x_* HTL with negligible ‐OH could protect the perovskite layer against moisture‐induced degradation.

Benefiting from the optimized film morphology, improved assembly regularity and preferred p‐type characteristics with QDs, the hole mobility of NiO*_x_* HTLs showed a more than threefold increase, from 3.98 × 10^−5^ to 1.29 × 10^−4^ cm^2^ V^−1^ s^−1^ (Figure 1f). Furthermore, the densely‐packed NiO*_x_* films also resulted in hydrophobicity improvement, as approved by increased water contact angle, and prolonged water dipping time (Figures S9 and S10, Supporting Information). The unencapsulated perovskite covered with QDs‐promoted NiO*_x_* film remained black even being directly dipped into water for 45 min, demonstrating outstanding stability against moisture. Therefore, schematics of NiO*_x_* films’ morphology were proposed in Figure 1g, intuitively showing that QD intercalation improved compactness of NiO*_x_* film on perovskite and increased hole extraction.

### 2.2 Enhancement in Hole Extraction from Perovskite to NiO*_x_* HTL By QD Intercalation

**Figure 2:**
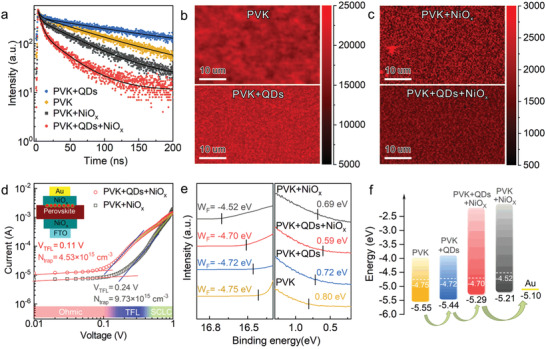
Surface passivation of perovskite and energy level adjustment between perovskite and NiO*_x_* with QD interlayer. a) TRPL spectra of PVK films with QDs, NiO*_x_* or both. b,c) Confocal PL intensity maps of the corresponding films. d) SCLC measurements of devices with the as‐drawn structure. e) UPS spectra of corresponding films. f) Schematic of energy band alignments of perovskite and NiO*_x_* without or with QDs. The white dotted lines refer to Fermi levels and the green arrows refer to hole transportation.

QD intercalation not only improved the hole mobility in NiO*_x_* HTLs, but also enhanced the hole extraction from PVK to NiO*_x_*, demonstrating the multiple roles of QDs in systematic interfacial engineering.  As shown in Figure S11 (Supporting Information), QDs mainly assembled at the grain boundaries of perovskite crystals. With the modification of QDs, the trap state density on perovskite film decreased and the charge recombination was suppressed, as demonstrated by steady‐state PL (Figure S12, Supporting Information) and time‐resolved PL spectra (**Figure** **2**a). The QDs‐covered PVK film showed a longer lifetime than the pristine PVK, indicating reduced nonradiative recombination centers for photoinduced charges in the former. The PVK film with NiO*_x_* exhibited shorter carrier lifetime after QD intercalation, indicating more efficient hole transfer. PL intensity mapping (Figure 2b) also confirmed the above result, showing that the emission distribution of PVK film was more uniform after QD intercalation. Moreover, the sample with QDs‐promoted NiO_x_ HTLs displayed more uniform intensity distribution and faster PL decay than the pristine one (Figure 2c). After QD intercalation, more effective hole extraction resulted from fewer nonradiative recombination centers, indicating less trap states for carriers at the interface between PVK and NiO*_x_*. Then, the measurement of space‐charge‐limited current (SCLC) was carried out to calculate the density of trap states (Figure 2d; Figure S13, Supporting Information). The trap‐state density showed nearly twofold decrease after QD promotion. Due to fewer trap states on QDs‐covered perovskite, the impedance for charge recombination was improved and the resistance for charge transfer was declined (Figure S14, Supporting Information). And the dark current density of the QDs‐promoted device was obviously lower than that of the control device, indicating that QDs prevented current leakage within the devices (Figure S15, Supporting Information).

Besides passivation effect of QDs on perovskite films, energy level alignments between each layer are an important factor in terms of charge transfer. The Fermi levels and the highest occupied molecular orbital (HOMO) levels of perovskite and NiO*_x_* films without or with QDs were measured by UPS spectra and Kelvin‐probe force microscopy (KPFM) (Figure 2e; Figure S16, Supporting Information). The energy fall between the HOMO level of PVK and HTL could result in an enormous interfacial charge recombination and a large *V*_OC_ loss in the whole device. The HOMO level of QDs‐covered perovskite layer (−5.44 eV) was higher than the pristine perovskite (−5.55 eV), while the HOMO level of QDs‐promoted NiO*_x_* HTL (−5.29 eV) was lower than the pristine NiO*_x_* deposited directly on perovskite (−5.21 eV). The shrunken energy gap would facilitate hole extraction through layers (Figure 2f). The Fermi level of NiO*_x_* also shifted from −4.52 to −4.70 eV upon QD intercalation, showing more p‐type property and thus higher hole mobility of QDs‐promoted NiO*_x_*. This was attributed to increased Ni^3+^/Ni^2+^ ratios in QDs‐promoted NiO*_x_* film as discussed above. The lower HOMO energy of QDs‐promoted NiO*_x_* contributed to a higher built‐in potential of the whole device, as tested with the Mott–Schottky plot (Figure S17, Supporting Information). As discussed above, QD intercalation was proved to facilitate hole transfer from PVK to NiO*_x_* and thus improve device's efficiency.

### 2.3 Overall Performance Boost of PSCs with QDs‐Promoted NiO*_x_* HTLs

**Figure 3:**
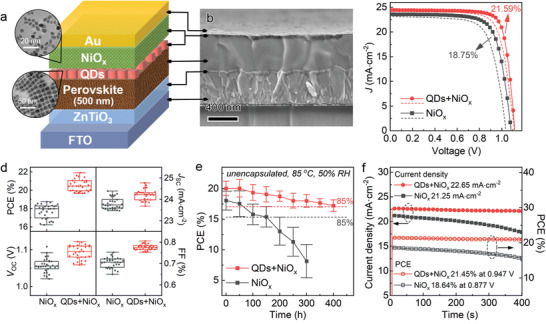
Performance enhancement of NiO*_x_*‐based PSCs with QD interlayers. a) Schematic of device configuration in this work and representative TEM images of NiO*_x_* NPs and CsPbI_1.85_Br_1.15_ QDs. b) Cross‐sectional SEM image of the PSC with QDs‐promoted NiO*_x_* HTL. c) *J–V* curves of the best NiO*_x_*‐based device without or with QDs (active area: 0.12 cm^2^). d) Storage stabilities of unencapsulated devices aged at 85 °C and 50% RH in air. e) Operational stabilities of the corresponding devices at MPP under 1‐sun illumination. f) Depth profiling of ToF‐SIMS for aged devices without or with QDs.

Based on the improvement in the properties of NiO*_x_* HTL and hole extraction between perovskite and NiO*_x_*, QD intercalation promoted NiO*_x_* NPs as effective io‐HTLs to boost the overall performance of n‐i‐p PSCs. The devices with structure of FTO/ZnTiO_3_/FA_0.85_MA_0.15_Pb(I_0.85_Br_0.15_)_3_/QDs/NiO*_x_*/Au were fabricated (**Figure** **3**a,b). The QD interlayer was too thin to be visible in the cross‐sectional scanning electron microscopy (SEM) image. Thus, cross‐sectional EDX mappings (Figure S18, Supporting Information) and depth profiling of time‐of‐flight secondary‐ion mass spectrometry (ToF‐SIMS) (Figure 3f) confirmed the existence of QDs between perovskite and NiO*_x_*. The independent peaks for Ni and Cs element in ToF‐SIMS demonstrated separate layers of QD interlayer and NiO*_x_* HTL. The device with pristine NiO*_x_* HTL was achieved by spin‐coating 15 mg mL^−1^ NiO*_x_* (dispersed in mixed chloroform and chlorobenzene) on perovskite without further annealing to reach the highest efficiency (Figure S19 and Table S1, Supporting Information). Surprisingly, the device with QDs‐promoted NiO*_x_* HTL exhibited a significant PCE enhancement and hysteresis reduction, with the top efficiency from 18.75% to 21.59% (Figure 3c; Table S2, Supporting Information). The current densities measured from *J–V* scan were verified by incident photons to current efficiency (IPCE) measurements, with each integrated *J*_SC_ deviating less than 5% from measured *J*_SC_ (Figure S20, Supporting Information). The highest PCE was achieved with QDs of CsPbI_1.85_Br_1.15_, after comparisons of QDs with varied chemical compositions (Figure S21, Supporting Information). The reproducibility was demonstrated from the sum of 30 devices for each type, with average PCE increasing from 17.71% (without QDs) to 20.52% (with QDs) (Figure S22, Supporting Information). Also, the devices with QDs‐promoted NiO*_x_* HTLs showed higher efficiency than other reported n‐i‐p devices with NiO*_x_* NPs and were even comparable to the fully developed p‐i‐n devices with NiO*_x_* NPs, as listed in Table S3 in the Supporting Information.

Besides efficiency improvement, the storage and operational stability of PSCs with QDs‐promoted NiO*_x_* HTLs were also enhanced. The heat/damp stabilities of unencapsulated devices were tested under harsh condition at 85 °C with 50% RH in ambient air. The unencapsulated PSCs with QDs‐promoted NiO*_x_* showed improved thermal and damp stability, remaining over 85% of original PCE after 400 h while the PCE of control device swiftly fell after 150 h (Figure 3d). NiO*_x_*‐based devices presented much better stability than the ones with common HTL, spiro‐OMeTAD (Figure S23, Supporting Information). Besides, operational stability of the devices were tracked at maximum power point (MPP). The device with QDs‐promoted NiO*_x_* exhibited a stable continuous output, indicating stable hole extraction therein (Figure 3e). To figure out the degradation process, 200‐h‐aged devices without or with QD intercalation was characterized and compared with fresh ones (Figure S24, Supporting Information). The results showed that the phase stability of perovskite in NiO*_x_*‐based devices was improved by QDs. Furthermore, the suppression of upward I^−^ migration was illustrated by ToF‐SIMS (Figure 3f), due to the denser film morphology of NiO*_x_* HTL with QD intercalation. In addition, cross‐sectional SEM‐EDX mapping and aging test in isopropanol further confirmed the improved stability of devices with QDs‐promoted NiO*_x_* HTLs (Figures S25 and S26, Supporting Information). The above results showed the advantages of QDs‐promoted NiO*_x_* PSCs in terms of both PCE and stability. The universality of QDs‐promoted NiO*_x_* HTLs were further verified in other perovskite systems like MAPbI_3_ (Figure S27 and Table S2, Supporting Information). The successful realization of QDs‐promoted NiO_x_ HTLs in n‐i‐p PSCs can compensate for the shortcomings of other HTLs in regular devices, as systematically analyzed in **Table** **1**.

**Table 1:** Advantages (√) and disadvantages (×) of each kind of HTLs for n‐i‐p PSCs

HTLs	PCE	Stability				Processability
		Thermal	Damp	Light	Electrode	
Organic materials	**√**	**×**	**×**	**×**	**×**	**√**
Cu‐based inorganic compounds	**√**	**√**	**√**	**√**	**×**	**×**
Sol–gel NiO*_x_*	**×**	**√**	**√**	**√**	**√**	**×**
Aqueous NiO*_x_* NPs	**×**	**√**	**√**	**√**	**√**	**×**
Previous nonaqueous NiO*_x_* NPs	**×**	**√**	**×**	**√**	**×**	**√**
**This work**: QDs‐promoted NiO*_x_* NPs	**√**	**√**	**√**	**√**	**√**	**√**

### 2.4 Practical Application of QDs‐Promoted NiO*_x_* HTLs in Large‐Area Modules and Semitransparent Devices

**Figure 4:**
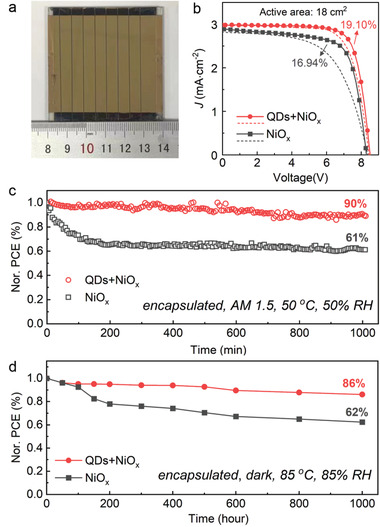
Performance improvement of large‐scale perovskite modules with QDs‐promoted NiO_x_ HTLs. a) Optical photo of a 36‐cm^2^ perovskite solar module with QD intercalation. b) *J–V* scans of modules of 18 cm^2^ active area with NiO*_x_* or QDs‐promoted NiO*_x_*. c) Operational stabilities of the corresponding encapsulated modules at MPP under 1‐sun illumination. d) Long‐term storage stabilities of encapsulated modules during 85 °C/85% RH aging.

After demonstrating the feasibility of NiO*_x_* HTLs promoted by QDs in small‐area PSCs, the application of QDs‐promoted NiO*_x_* HTLs was further extended to opaque large‐scale perovskite solar modules and semitransparent small solar devices, showing great potential for future commercial manufacture. QDs‐promoted NiO*_x_* has been proved to form uniform and pin‐hole‐free films on perovskites at a scale of 36 cm^2^ (Figure S28, Supporting Information), ready for solar module fabrication. Then 36‐cm^2^ solar modules (18‐cm^2^ aperture area) consisting of 8 separate cells in series were fabricated with NiO*_x_* HTLs (**Figure** **4**a; Figure S29, Supporting Information). QD intercalation endowed NiO*_x_*‐module with increased PCE (from 16.94% to 19.10%) (Figure 4b; Table S4, Supporting Information). The encapsulated QD‐promoted NiO*_x_*‐based module also exhibited improved operational and storage stability. The continuous operational stabilities of encapsulated modules were tracked at MPP output in ambient air (≈50 °C/50% RH) under 1‐sun illumination. The QDs‐promoted module showed significant improvement in working stabilities as compared with the control module during 1000‐min tracking (Figure 4c). Furthermore, the storage stabilities of encapsulated modules under extreme condition were tested at 85 °C, 85% RH for 1000 h (Figure 4d). The module with pristine NiO*_x_* showed a rapid decay in the first 200 h, and maintained only 62% of the initial efficiency after 1000‐h aging. The device with QDs‐promoted NiO*_x_* showed better stability with 86% of PCE left after 1000 h, benefiting from the ordered and denser NiO*_x_* film with fewer hydroxy groups. The improvement in long‐term stability of PCE with QD intercalation mainly lied in the enhanced stability of fill factor (Figure S30, Supporting Information). As far as we know, the HTLs that can resist aging at 85 °C/ 85% RH are rare in n‐i‐p devices.^[^ ^23^ ^]^ Thus, the development of NiO*_x_* as stable io‐HTL for n‐i‐p devices is of great significance for the manufacture of commercially viable solar modules in the future.

**Figure 5:**
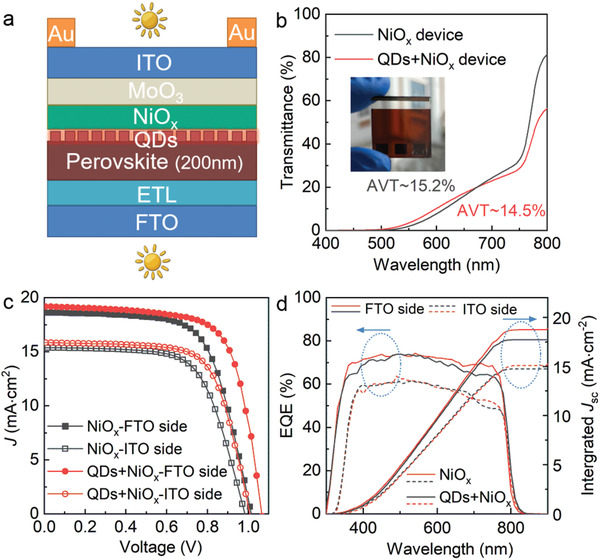
Performance development of semi‐transparent NiO*_x_*‐based solar devices. a) Schematic of semitransparent device with n‐i‐p configuration. b) Transmittance spectra and photo (inset) of the full ST‐devices. c) *J–V* curves of ST‐devices illuminated from both sides. d) IPCE plots and integrated *J*_SC_ for the corresponding ST‐devices.

In addition, inspired by high transmittance of NiO*_x_* between 300 and 800 nm (light absorption range of perovskite films), QDs‐promoted NiO*_x_* was extended to the fabrication of semitransparent PSCs (ST‐PSCs), which have great potential in building‐integrated photovoltaics and tandem cells. Sectional configuration of the ST‐device was presented in **Figure** **5**a, with transparent conducting oxide as transparent electrodes at both sides. The transmittance of NiO*_x_* was higher than spiro‐OMeTAD (Figure S31, Supporting Information). After deposition of MoO_3_/ITO as transparent top electrode, the transmittance of NiO*_x_* film decreased from ≈95% to ≈76%. The introduction of QD interlayer had no obvious effect on transmittance. To ensure transparency of the whole ST‐devices, relatively thin perovskite films were also required. FAMAPb(IBr)_3_ perovskites with thickness at around 200 nm were deposited, using the same spin‐coating method as the regular perovskite films but lower Pb^2+^ (0.75 m) in the perovskite precursor. To verify film quality of the ultrathin perovskite layer, devices using opaque Au electrode and 200‐nm perovskite were also fabricated (Figure S32 and Table S5, Supporting Information). Benefited from ultra‐thin perovskite films, the average visible transparencies (AVTs) of the fabricated ST‐devices with NiO*_x_* and QDs‐promoted NiO*_x_* were 15.2% and 14.5%, respectively (Figure 5b). The cross‐sectional SEM of the ST‐device with QDs‐promoted NiO*_x_* was shown in Figure S33 in the Supporting Information. The pristine NiO*_x_*‐based ST‐device showed efficiencies of 12.40% and 9.83% illuminated from the FTO side and ITO side, respectively (Figure 5c; Table S6, Supporting Information). The QDs‐promoted one had higher efficiencies of 14.25% (FTO side) and 10.96% (ITO side). The *J*_SC_ data measured from *J–V* scan were also confirmed by IPCE, from which the integrated *J*_SC_ had no more than 5% variation (Figure 5d; Table S7, Supporting Information). The above results indicate excellent compatibility of NiO*_x_*‐based HTLs with semitransparent solar devices, and show great potential for manufacturing next generation photovoltaic devices.

## 3 Conclusion

In conclusion, NiO*_x_*‐based HTLs have been successfully promoted by QD intercalation and applied in n‐i‐p PSCs. The QD intercalation between perovskites and NiO*_x_* was found to improve hole mobility within NiO*_x_* films by enhancing film morphology and steric regularity of NiO*_x_* films, and facilitate hole extraction from perovskites to HTLs by passivating defects and optimizing HOMO level alignments. QDs‐promoted NiO*_x_* HTLs helped to fabricate n‐i‐p devices with high efficiency and distinguished stability, showing great potential in fabrication of large‐scale devices and semitransparent devices. Through systematic interfacial engineering by multifunctional QDs, this work paves the way for the commercialization of n‐i‐p perovskite photovoltaics based on NiO*_x_* or other p‐type nanoparticle HTLs.

## 4 Experimental Section

### Synthesis Methods

NiO*_x_* nanoparticles were synthesized using a noninjection method as previously reported.^[^ ^24^ ^]^ The reaction mixture containing nickel stearate (3 mmol, Sinopharm Chemical Regent Co., Ltd.), lithium stearate (1.2 mmol, Sinopharm), 1‐octadecanol (18 mmol, Alfa Aesar) and 1‐octadecene (ODE, 30 mL, Alfa Aesar) was added to a two‐neck flask under nitrogen and dissolved at 80 °C. Then the system was sealed and heated at 245 °C for 3 h. After the reaction was cooled to room temperature, the product was collected by adding a mixture of acetone (20 mL, Sinopharm) and ethyl acetate (20 mL, Sinopharm), and further purified using the combination of hexane (Sinopharm) and ethanol (Sinopharm). Finally, the product was dispersed in hexane and left overnight. The insoluble precipitate was removed by centrifugation. NiO*_x_* nanoparticles were extracted by adding ethanol to the liquid supernatant. After centrifugation, the final product was dried under vacuum.

CsPbI*_x_* Br_1−_ *_x_* quantum dots were synthesized by the commonly‐used one‐pot hot‐injection method.^[^ ^25^ ^]^ Cs_2_CO_3_ (0.4 g, Tokyo Chemical Industry Co., Ltd. (TCI)), ODE (40 mL), and oleic acid (2.5 mL, TCI) were loaded into 100 mL 3‐neck flask and the mixture was dissolved under a vacuum at 120 °C for 0.5 h. Then, the flask was refilled with N_2_ and preheated to 70 °C. In another pot, different ratios of PbBr_2_ (TCI) and PbI_2_ (Xi'an Baolaite Corp), which is defined by chemical formula of CsPbI*_x_* Br_1−_ *_x_* QDs, were added together with 1‐octadecene (the concentration of Pb^2+^ was 1 m). The mixtures were degassed under a vacuum (≈0.1 Torr) at 120 °C for 1 h and excess oleic acid (10 mL) and oleylamine (10 mL, Aladdin) (both preheated at ≈70 °C) were injected. After PbBr_2_ and PbI_2_ were completely dissolved, the Cs‐oleate compound was swiftly injected into the reaction mixture at 175 °C. After the reaction mixture was quenched by an ice bath (≈5 s after injection), methyl acetate (MeOAc, 70 mL, ultradry, Aladdin) was added into the supernatant to precipitate QDs. Then the wet pellets of QDs were washed with hexane twice and precipitated again with an equal volume MeOAc. After that, the remained solids were dried in a vacuum at room temperature to obtain QDs with uniform sizes.

### Device Fabrication

0.12‐cm^2^ perovskite solar cell: First, ZnTiO_3_ (ZTO) electron transport layer was deposited on clean and patterned 2 × 2 cm^2^ FTO by spray coating the mixture solution of Zn(OAc)_2_ (2.5 mL, 0.2 m in ethanol, Sinopharm), titanium diisopropoxide bis(acetylacetonate) (0.5 mL, 75% in isopropanol, Alfa Aesar), and thiourea (4.7 mg) in ethanol (50 mL) at 450 °C for 30 min, as reported in our previous work.^[^ ^26^ ^]^ Then, perovskite films with different compositions were deposited by spin‐coating methods. FA_0.85_MA_0.15_PbI_2.55_Br_0.45_: A solution of 900 mg FAPbI_3_, 22.5 mg MAPbBr_3_, 65 mg PbI_2_, 33 mg MACl dissolved in 1 mL DMF/DMSO (4/1) was spun at 1200 rpm for 5 s followed by 6000 rpm for 20 s, with diethyl ether sprayed at 5 s prior the end of the process. Then the wet film was annealed at 110 °C for 20 min. MAPbI_3_: A solution of 620 mg MAPbI_3_ in 600 µL DMF/DMSO (4/1) was spun at 4000 rpm for 20 s and diethyl ether was dropped at the first 8 s after spin‐coating began, followed by annealing at 100 °C for 10 min. For NiO*_x_* as hole transport layer, presynthesized powders of oleophilic NiO*_x_* NPs were dissolved into chloroform/chlorobenzene (2/1) and spin‐coated onto perovskite film. For QD intercalation, 5 mg mL^−1^ QDs in octane was spin‐coated onto perovskite films, followed by NiO*_x_* deposition. For spiro‐OMeTAD as HTL, spiro‐OMeTAD dissolved in chlorobenzene (80 mg mL^−1^) with 9.1 mg Li‐TFSI and 29 µL *t*BP, followed by spin‐coating at 3500 rpm. Then 60‐nm Au electrode was evaporated using thermal evaporation system (Angstrom, SQC‐310C) at the speed of 2.5 Å s^−1^ under 3.7 × 10^−7^ vacuum degree with mask of 0.12 cm^2^ active area.

36‐cm^2^ perovskite solar module—6 × 6 cm^2^ FTO substrate was scribed into eight separated cells by laser (P1 line). The coating process for each layer was the same as small‐area solar cells except for the laser scribing patterning procedure. 280 µm P2 line was scribed after perovskite and NiO*_x_* deposition, using laser with pulse energy of 30 µJ, spot size of 200 µm, and pulse frequency of 30 kHz. 400 µm P3 line was scribed after Au evaporation, using 30 µJ pulse energy. The active area of a 6 × 6 cm^2^ module was 18 cm^2^. For the module encapsulation, fabricated module was sealed with a cover glass and polyisobutylene under vacuum, with pressure of 300–400 mTorr for 10 min at 90 °C.

0.12‐cm^2^ semitransparent perovskite solar cell—The deposition of each layer was the same with opaque PSCs on 2 × 2 cm^2^ FTO. The transparent electrode with construction of MoO_3_/ITO/Au grid was fabricated layer by layer on perovskite/NiO*_x_* film. 15 nm MoO_3_ was thermal evaporated with the speed of 1 Å s^−1^ under 5 × 10^−7^ vacuum degree to protect the film from sputtering energy. 150 nm ITO was RF‐sputtered with RF magnetron sputtering (Shenyang Kejing, VTC‐600‐2HD) from a ceramic 2‐inch ITO target (90% wt In_2_O_3_ and 10% wt SnO, Hefei Kejing) at a pressure of 2 mTorr. The RF‐power was maintained at 70 W and the substrate temperature was 70 °C during the 60 min deposition process. 80 nm gold grid was thermally evaporated through a shadow mask to collect the charge carriers and improve conductivity of ITO electrode.

### Characterization Methods

Current–voltage characteristics (*J–V* plots) were recorded on a solar simulator equipped with a Keithley 2400 source meter and a 300‐W collimated xenon lamp (Newport) calibrated with a light intensity to 100 mW cm^−2^ at AM 1.5 G solar light conditions by a standard silicon solar cell. The incident photon‐to‐electron conversion efficiencies (IPCEs) were measured on an external quantum efficiency (EQE) system (Newport) by focusing a monochromatic beam of light onto the devices. The X‐ray diffraction (XRD) patterns were analyzed on an X‐ray diffraction meter (Rigaku, Ultima IV) with a Cu K_*α*_ radiation source. Small angle X‐ray scattering (SAXS) spectra were carried out with an Anton‐paar SAXSess mc^2^. X‐ray photoelectron spectroscopy (XPS) spectra were recorded by Thermo Scientific ESCALAB Xi+. An Al K_*α*_ (1486.6 eV) X‐ray was used as the excitation source. All data were calibrated by C 1s at 284.6 eV. Ultraviolet photoelectron spectroscopy (UPS) was obtained at the same equipment using He (21.2 eV) source with a bias voltage of 5 eV. Scanning electron microscope (SEM) images were recorded using Zeiss Gemini SEM 500 under 5 kV. Energy‐dispersive X‐ray spectroscopies (EDX) were carried out on the same instrument with Ultim Extreme detector under 7 kV. Transmission electron microscopy (TEM) images were carried out on Tecnai F‐30 operated at 300 kV. Thermogravimetric analysis (TGA) spectra were measured with Netzsch, STA449F5. Fourier transform infrared (FTIR) spectra were recorded on a Bruker Vertex 70 V spectrophotometer at room temperature. AFM and KPFM images were obtained by an atomic force microscope (Oxford Instruments Asylum Research Cypher ES) using a conducting probe whose surface potential is calibrated with a fresh Au film. Element analysis of NiO*_x_* powder was conducted on Elementar Analysensyetem GmbH, Vario EL III. UV–vis absorption spectra were measured on shimadzu UV‐2600. The steady‐state photoluminescence spectra (PL) and time‐resolved photoluminescence (TRPL) spectra were carried on an Edinburgh Instruments FLS1000 spectrometer. The PL mapping was performed on a laser Raman microscope (RAMAN‐11, Nanophoton). The electrochemical impedance spectra (EIS) and the maximum power point (MPP) outputs of the devices were executed on an electrochemical workstation (CHI 760E), under AM 1.5G illumination. The Mott–Schottky plots of the devices were measured on CHI 760E with 1000 Hz frequency and without illumination. Measurements of space‐charge‐limited current (SCLC) and charge mobility were also carried out with CHI 760E. SCLC spectra were measured with capacitor‐like devices with perovskite films sandwiched between sol–gel‐deposited NiO*_x_* and oleophilic NiO*_x_* NPs. The defect density was calculated according to the equation *N*_trap_ = 2*ε*⋅*ε*_0_⋅*V*_TFL_/*e*⋅*L* ^2^, where *ε* and *ε*_0_ are the dielectric constants of the perovskite and vacuum permittivity, respectively, *L* is the thickness of obtained perovskite film and *e* is the elementary charge. The hole mobilities were calculated from the Mott–Gurney law by the equation: *J* = 8/9 × *ε*⋅*ε*_0_⋅*µ*⋅*V* ^2^/*L* ^3^, where *ε* and *ε*_0_ are the dielectric constants, *µ* is the hole carrier mobility, *V* is the voltage drop across the device and *L* is the thickness of NiO_x_. Time‐of‐flight secondary ion mass spectrometry (ToF‐SIMS) was performed on Model TOFSIMS 5 (Münster) with the pulsed primary ions from O_2_ liquid‐metal ion gun (1 keV) for sputtering and Bi^+^ pulsed primary ion beam for analysis (30 keV). The sputtering area was 200 × 200 µm^2^ and the analysis area was 94 × 94 µm^2^.

## Conflict of Interest

The authors declare no conflict of interest.

## Supporting information

Supporting Information

## Data Availability

The data that support the findings of this study are available from the corresponding author upon reasonable request.
