# 85 °C/85%‐Stable n‐i‐p Perovskite Photovoltaics with NiO_*x*_ Hole Transport Layers Promoted By Perovskite Quantum Dots

**DOI:** 10.1002/advs.202206766

**Published:** 2023-01-13

**Authors:** Fangwen Cheng, Fang Cao, Binwen Chen, Xinfeng Dai, Ziheng Tang, Yifei Sun, Jun Yin, Jing Li, Nanfeng Zheng, Binghui Wu

*Adv. Sci*. **2022**, *9*, 2201573

DOI: 10.1002/advs.202201573

In the original published article, Figure 3 is incorrect. Please find the correct Figure 3 below. The authors apologize for any inconvenience caused.

**Figure 3:**
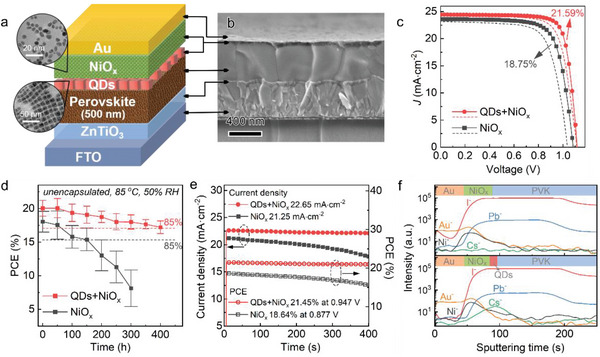
Performance enhancement of NiO_*x*_‐based PSCs with QD interlayers. a) Schematic of device configuration in this work and representative TEM images of NiO_*x*_ NPs and CsPbI_1.85_Br_1.15_ QDs. b) Cross‐sectional SEM image of the PSC with QDs‐promoted NiO_*x*_ HTL. c) *J–V* curves of the best NiO_*x*_‐based device without or with QDs (active area: 0.12 cm^2^). d) Storage stabilities of unencapsulated devices aged at 85 °C and 50% RH in air. e) Operational stabilities of the corresponding devices at MPP under 1‐sun illumination. f) Depth profiling of ToF‐SIMS for aged devices without or with QDs.

