# Supplementary material for: 85 °C/85%‐Stable n‐i‐p Perovskite Photovoltaics with NiO x Hole Transport Layers Promoted By Perovskite Quantum Dots
Source: Adv Sci (Weinh). 2022 Jul 20;9(26):2201573. doi: 10.1002/advs.202201573 (PMC9475515; doi:10.1002/advs.202201573)
Supplement: Supplementary file 1 — Supporting Information [file ADVS-9-2201573-s001.pdf]

ADVANCED SCIENCE
Open Access

Supporting Information

for *Adv. Sci.*, DOI 10.1002/advs.202201573

85 °C/85%-Stable n-i-p Perovskite Photovoltaics with NiO_x Hole Transport Layers Promoted By Perovskite Quantum Dots

*Fangwen Cheng, Fang Cao, Binwen Chen, Xinfeng Dai, Ziheng Tang, Yifei Sun, Jun Yin, Jing Li, Nanfeng Zheng and Binghui Wu**

Supporting information for

85 °C/85%-Stable n-i-p Perovskite Photovoltaics with NiO_x Hole Transport Layers Promoted by Perovskite Quantum Dots

*Fangwen Cheng,† Fang Cao,† Binwen Chen, Xinfeng Dai, Ziheng Tang, Yifei Sun, Jun Yin, Jing Li, Nanfeng Zheng, Binghui Wu**

State Key Laboratory for Physical Chemistry of Solid Surfaces, Collaborative Innovation Center of Chemistry for Energy Materials (iChEM), National & Local Joint Engineering Research Center of Preparation Technology of Nanomaterials, College of Chemistry and Chemical Engineering, Pen-Tung Sah Institute of Micro-Nano Science and Technology, College of Energy, Jiujiang Research Institute, Innovation Laboratory for Sciences and Technologies of Energy Materials of Fujian Province (IKKEM), Xiamen University, Xiamen 361005, China.

[†] These authors contributed equally to this work.

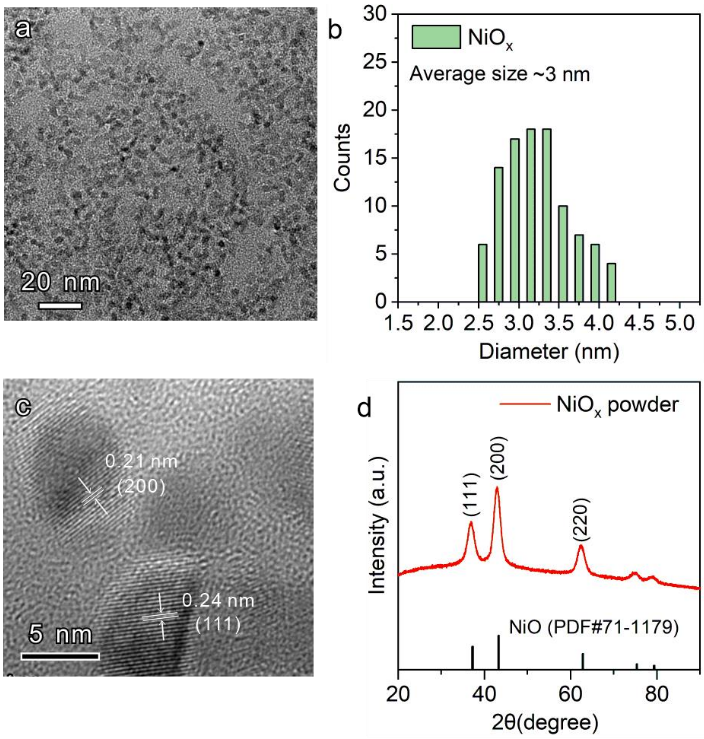

Figure S1. Characterizations of NiO_x NPs. (a) TEM image of as-synthesized NiO_x NPs. (b) Statistics for diameters of NiO_x NPs summarized from 100 samples. (c) High-resolution TEM image of isolated particles. The inter-planar distances of 0.21 and 0.24 nm can be indexed to (200) and (111) planes of NiO_x, respectively. (d) XRD of NiO_x NPs.

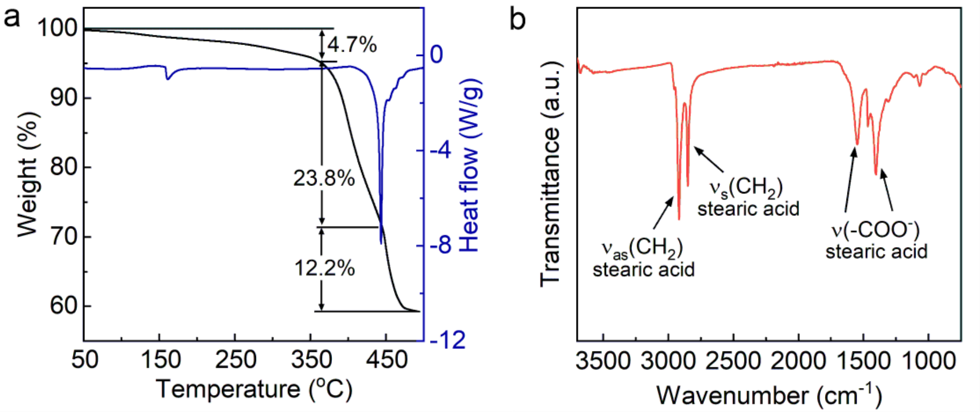

Figure S2. Characterizations of ligands on NiO_x NPs. (a) TG curves of NiO_x NPs. The mass ratio of ligands on NiO_x was 36%. (b) FTIR spectrum of NiO_x NPs.

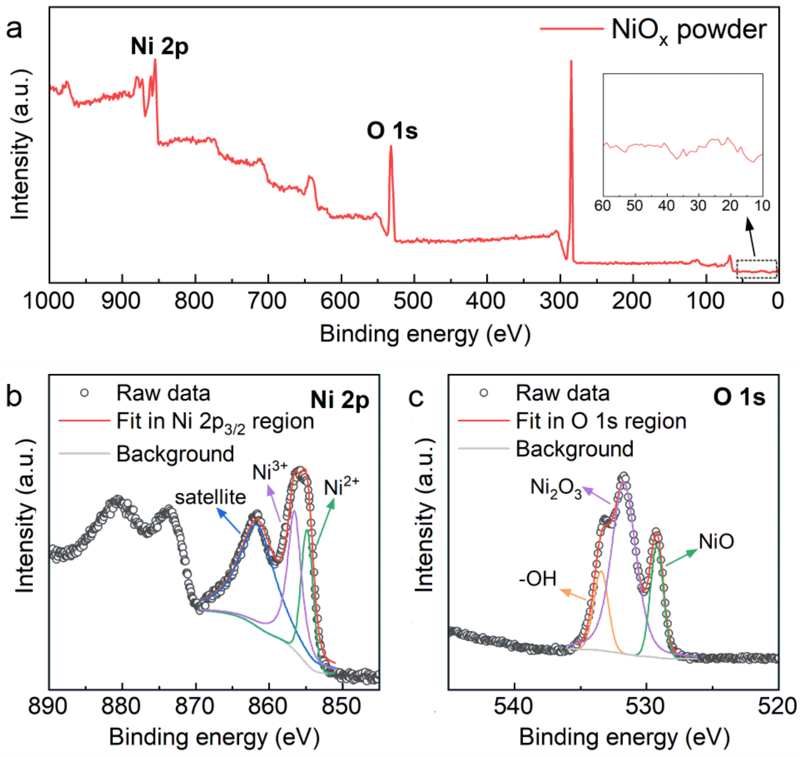

Figure S3. Electronic states of NiO_x NPs. (a) Full XPS spectrum of NiO_x NPs. The signal for Li 1s with binding energy at around 55 eV was not detected. Magnified XPS of (b) Ni 2p and (c) O 1s of NiO_x NPs.

Characterization of NiO_x nanoparticles. Firstly, NiO_x NPs were synthesized with stearic-acid ligands to ensure that they can be dispersed in non-polar solvents and deposited directly onto perovskite thin films with high chemical compatibility. The following features of NiO_x NPs were demonstrated to ensure that NiO_x NPs were ready for HTL usage in n-i-p PSCs: 1) monodispersity in non-polar solvents, 2) uniform and small sizes, 3) isotropy in morphology, 4) high crystallinity, 5)

appropriate ligand amount and 6) p-type chemical component. TEM images showed that the spherical NiO_x NPs possess a uniform particle size of about 3 nm with well-resolved lattice fringes, and XRD patterns of NiO_x indicated a rock salt crystalline structure (Figure S1). Figure S2 indicated the stability of NiO_x NPs with a large temperature window (< 350 ^{o}C). The ligand amount of NiO_x was calculated to be ~30-40%, corresponding with elemental analysis result (mass ratio of C at 29%). And FTIR spectrum of NiO_x NPs showed typical peaks for stearic acid, identifying the ligands on NiO_x NPs. In addition, although the synthesis process using nickel stearate and lithium stearate as reaction precursors, the synthesized NiO_x NPs contain negligible Li (as shown in full XPS spectrum, Figure S3a). The XPS of Ni 2p and O 1s of NiO_x illustrated that Ni(II) and Ni(III) coexisted in the as-synthesized NiO_x NPs (Figure S3b-c). And the existence of Ni(III) endows NiO_x NPs with oxygen vacancies and p-type property. The above features of NiO_x NPs are requisite for low-temperature deposition as HTL on perovskite films.

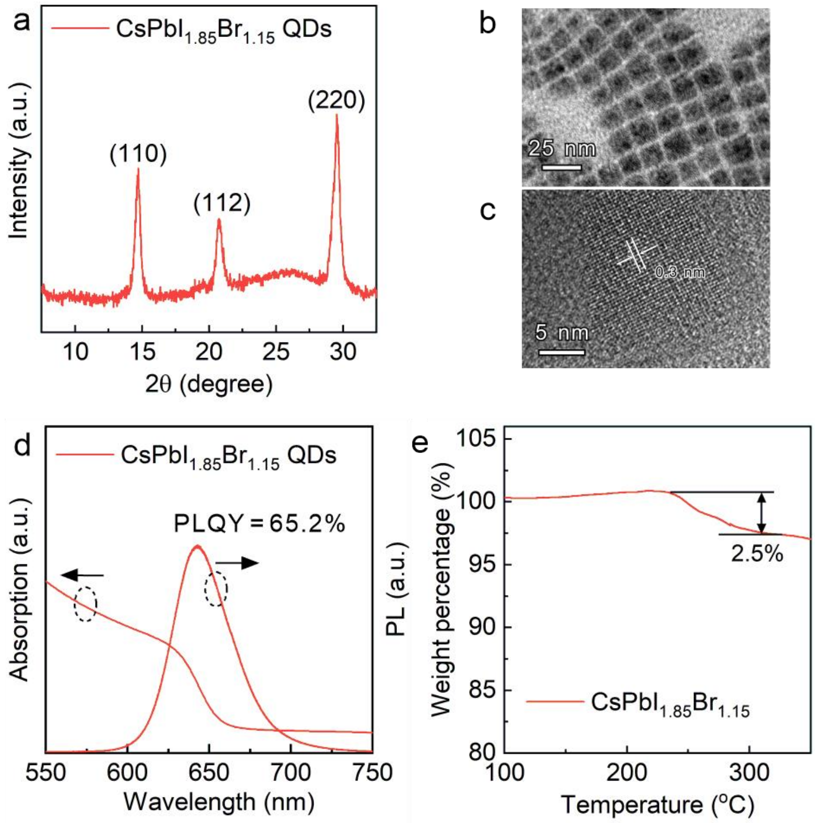

Figure S4. (a) XRD pattern of as-synthesized perovskite QDs with chemical formula of $CsPbI_{1.85}Br_{1.15}$. The peaks arose at 14.5°, 20.3°, and 29.2°, indicating the cubic α-phase of QDs. (b) Typical TEM image and (c) high-resolution TEM image of QDs. (d) UV-vis absorption and PL emission of QDs. The absorption onset demonstrated that the bandgap of QDs were 1.89 eV and the sharp PL emission showed the uniform size and pure phase of QDs. (e) TG curve of QDs, showing the amounts of oleic ligands on QDs were ~2.5%, corresponding with the reported value.[1]

Characterization of $CsPbI_xBr_{1-x}$ quantum dots. Perovskite QDs with chemical formula of $CsPbI_xBr_{1-x}$ and particle size of around 15 nm were synthesized using the hot injection method, of which the composition can be adjusted according to the precursor ratios. The synthesizing and washing steps of QDs were the same as our previously reported work. The successful synthesis of QDs with cubic phase, uniform size and moderate amount of ligand was confirmed by XRD pattern, TEM image, UV-vis absorption spectrum and PL emission spectrum (Figure S2). And QDs can be monodispersed in non-polar solvents like octane, which was suitable for the modification on perovskite films with high chemical compatibility.

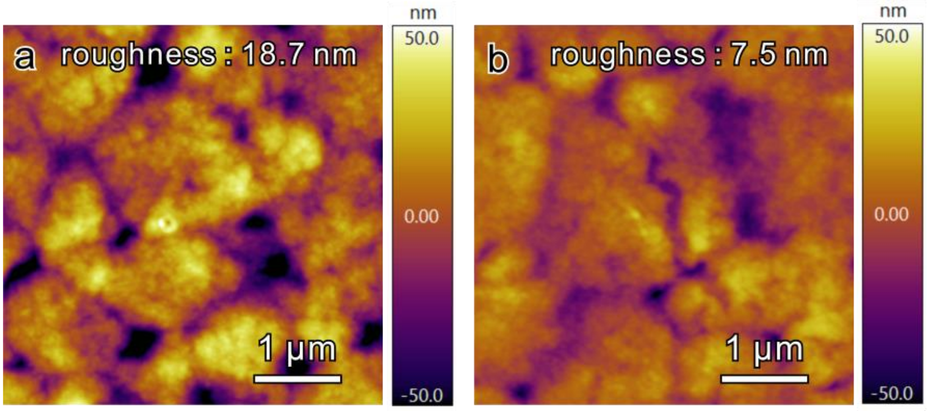

Figure S5. AFM images of NiO_x films on bare perovskite or QDs-covered perovskite.

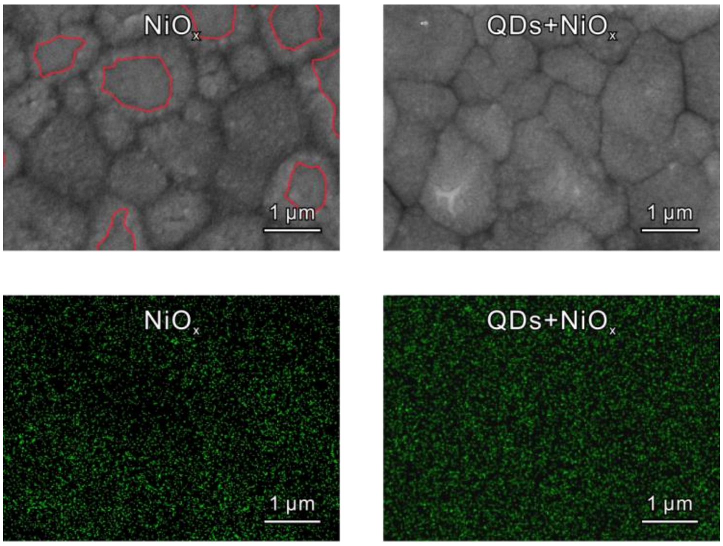

Figure S6. SEM images and EDX mappings of NiO_x and QDs-promoted NiO_x.

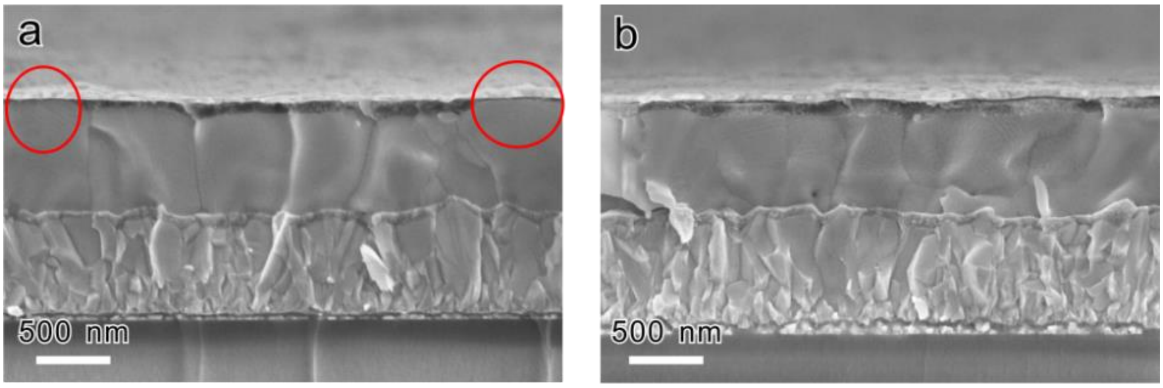

Figure S7. Cross-section SEM images of NiO_x-devices (a) without or (b) with QD intercalation. NiO_x film in the pristine device showed uneven morphology, with Au electrode contact directly with perovskite film in some areas, causing stability concerns. While in the promoted device, NiO_x covered uniformly on the QDs-covered perovskite.

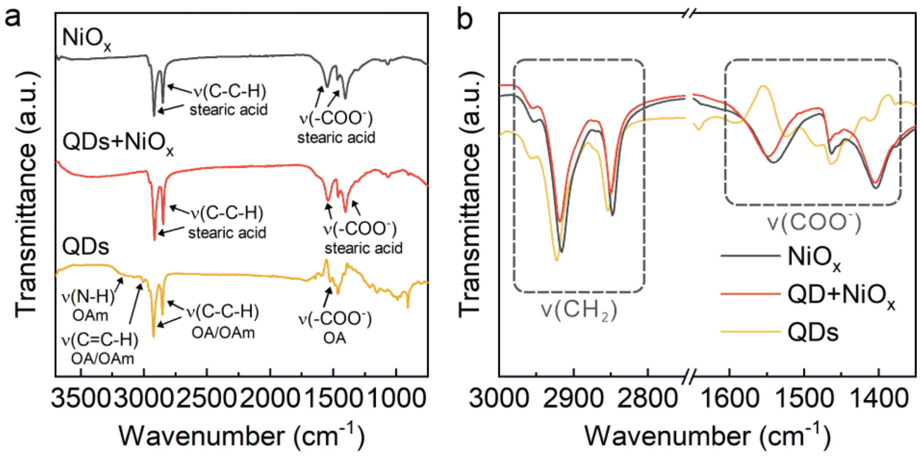

Figure S8. (a) FTIR spectra of NiO_x, QDs-promoted NiO_x and pure QDs. (b) Magnified spectra for vibration peaks of $-CH_2$ and $-COO^-$ in the corresponding samples.

Pristine NiO_x and QDs-promoted NiO_x showed typical peaks for C-H vibrations of $-CH_2$ (~2850 and ~2920 cm^{-1}) and peaks for strenching vibration of $-COO^-$ (~1400 and 1550 cm^{-1}) from stearate ions. The spectrum of pure QDs showed peaks for oleyl ions. The vibration peaks for $-CH_2$ and $-COO^-$ of stearate ligands on NiO_x were both red shifted after QD promotion. The results indicated that QD intercalation influenced the self-assembly behavior of NiO_x NPs with interaction between oleyl ligands on QDs and stearate ligands on NiO_x. And QD promotion affected binding states of stearate ligands on NiO_x core shell, altering electronic status of Ni^{2+}/Ni^{3+}.

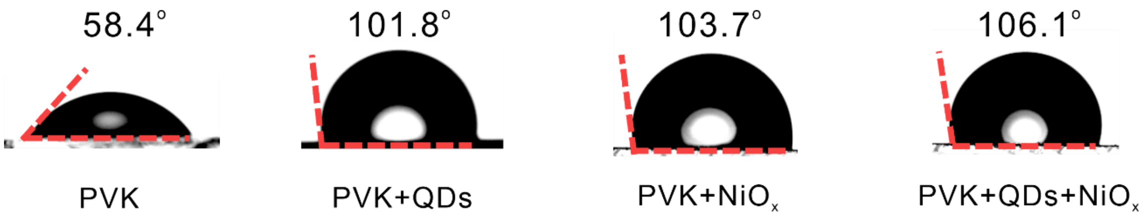

Figure S9. Water contact angle data of various films.

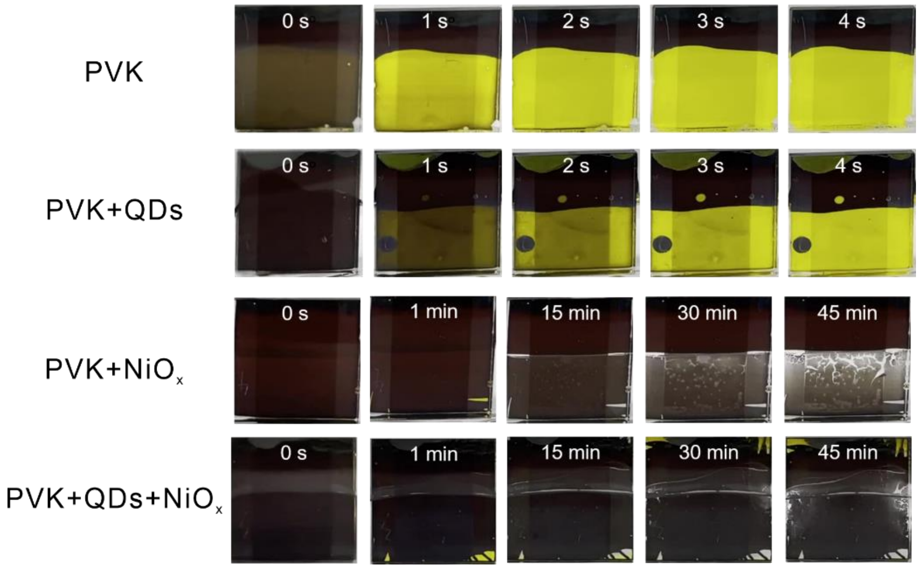

Figure S10. Photographs of various unencapsulated films being dipped into water for different time.

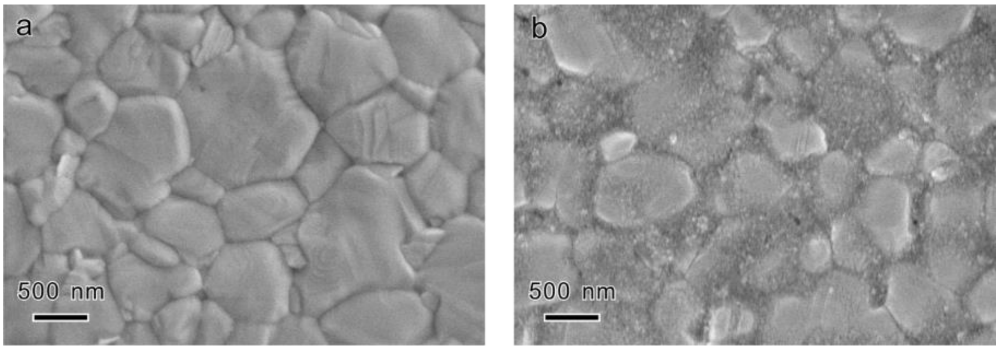

Figure S11. SEM images of (a) pristine perovskite surface and (b) perovskite covered with 5 mg/mL QDs.

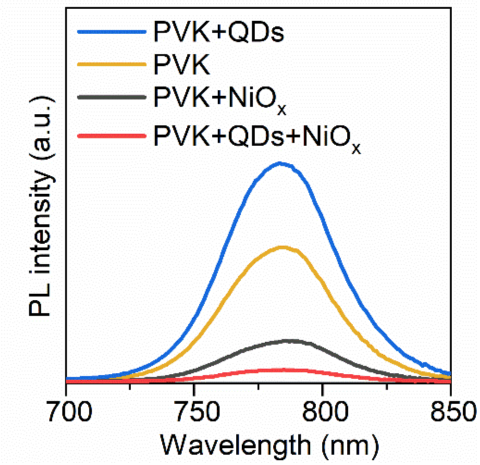

Figure S12. Steady PL emission spectra of various films.

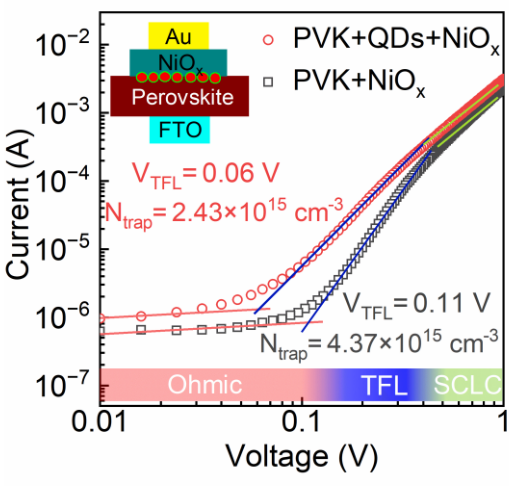

Figure S13. SCLC measurements of the control NiO_x-device and the QD-promoted NiO_x device with the as-drawn structure (FTO/perovskite/QDs/NiO_x/Au).

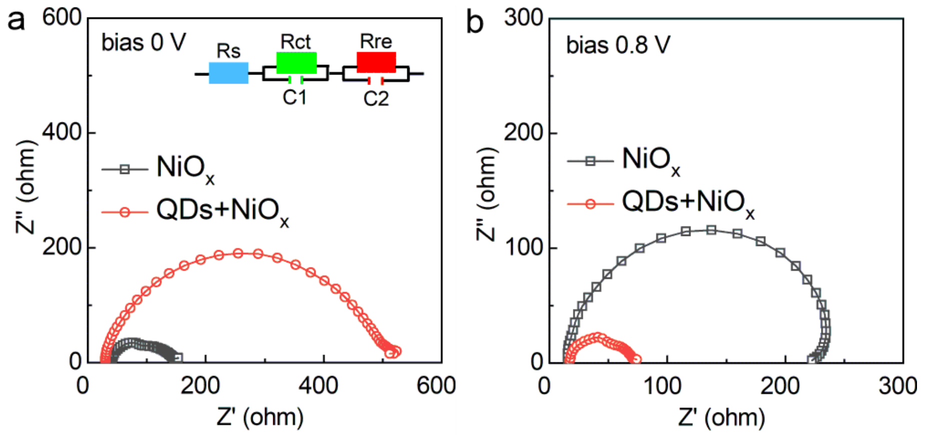

Figure S14. Nyquist plots under 1-sun illumination of NiO_x-devices without or with QDs at a bias voltage of (a) 0 V or (b) 0.8 V. The 0-V impedance showed the charge recombination rate in the perovskite and at the interface, while the 0.8-V impedance referred to the charge transfer rate along the whole device.

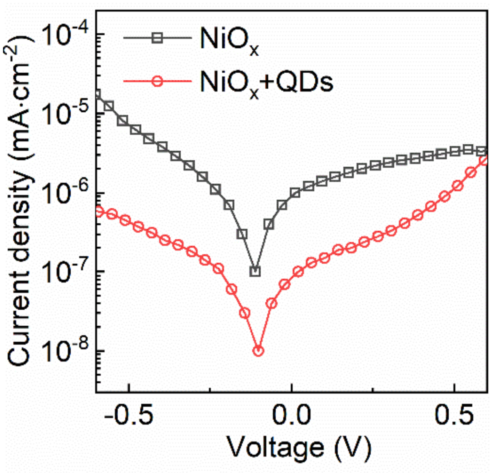

Figure S15. *J-V* scans under dark environment of NiO_x-devices without or with QDs.

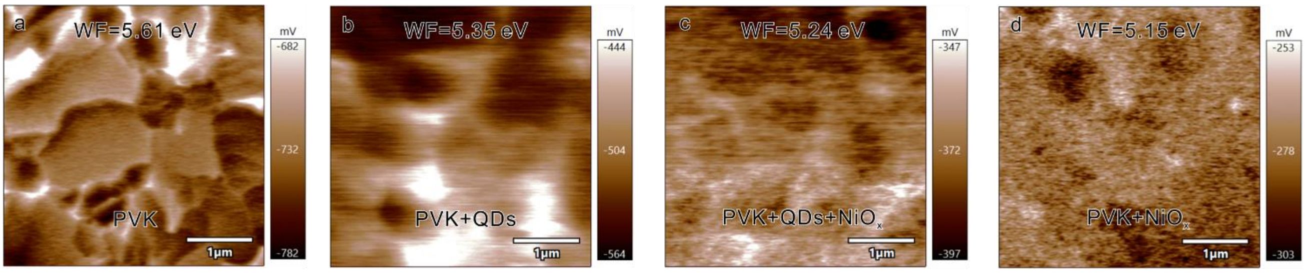

Figure S16. KPFM images of (a) pristine perovskite, (b) QDs-covered perovskite, (c) NiO_x on QDs-covered perovskite and (d) NiO_x on pristine perovskite.

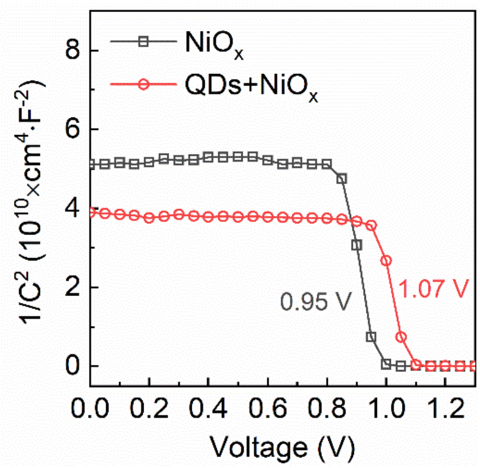

Figure S17. Mott-Schottky plots of NiO_x-devices without or with QDs.

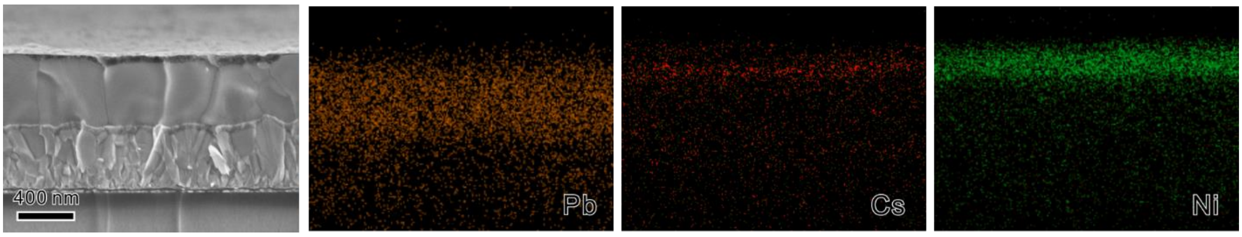

Figure S18. SEM image and EDX mappings of cross section of the device with QDs-promoted NiO_x HTL.

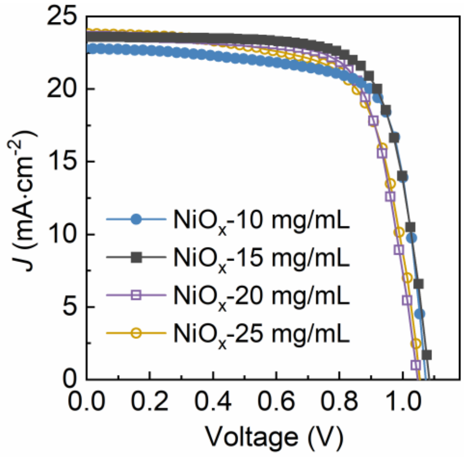

Figure S19. *J-V* curves of devices with structure of FTO/ZTO/FAMAPb$(IBr)_3$/NiO_x/Au. Each NiO_x was deposited from their solution in the mixed solvent of chloroform/chlorobenzene with different concentrations.

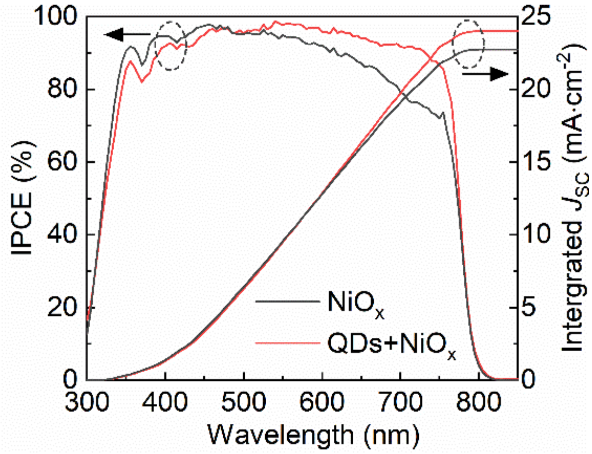

Figure S20. IPCE curves and integrated J_{SC} of NiO_x-based devices without or with QD promotion. The integrated J_{SC} for the NiO_x-devices without and with QDs were 22.75 and 24.05 mA·cm^{-2}, respectively, in accordance with the current density measured from *J-V* scan under 1-sun illumination.

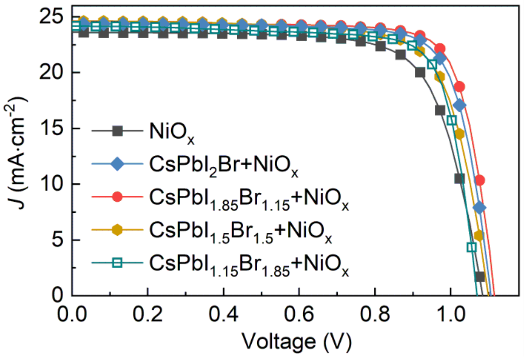

Entry	J_{sc} [mA·cm^{-2}]	V_{oc} [V]	FF [%]	PCE [%]
w/o QDs	23.62	1.083	73.32	18.75
$CsPbI_2Br$	24.40	1.104	78.07	21.03
$CsPbI_{1.85}Br_{1.15}$	24.86	1.114	79.29	21.96
$CsPbI_{1.5}Br_{1.5}$	24.64	1.097	74.57	20.15
$CsPbI_{1.15}Br_{1.85}$	24.17	1.068	78.27	20.20

Figure S21. Comparison of PCEs of NiO_x-based PSCs with different composition of $CsPbI_xBr_{1-x}$ QDs. The $CsPbI_{1.85}Br_{1.15}$ QDs endowed the best performance of PSCs and were mostly used in this work.

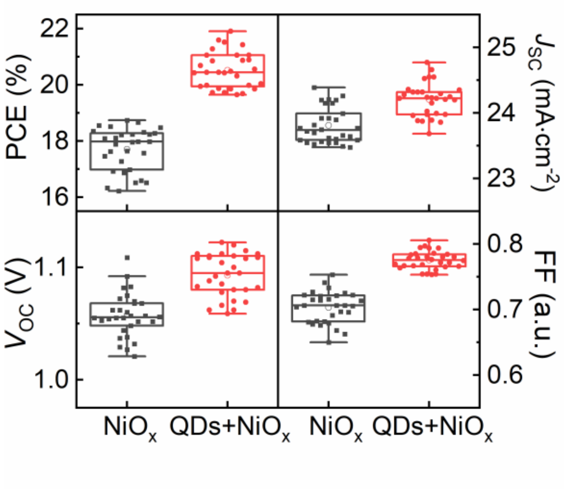

Figure S22. Statistical data for PCE, V_{OC}, J_{SC} and fill factor of 30 devices with NiO_x HTL and QDs-promoted NiO_x HTL.

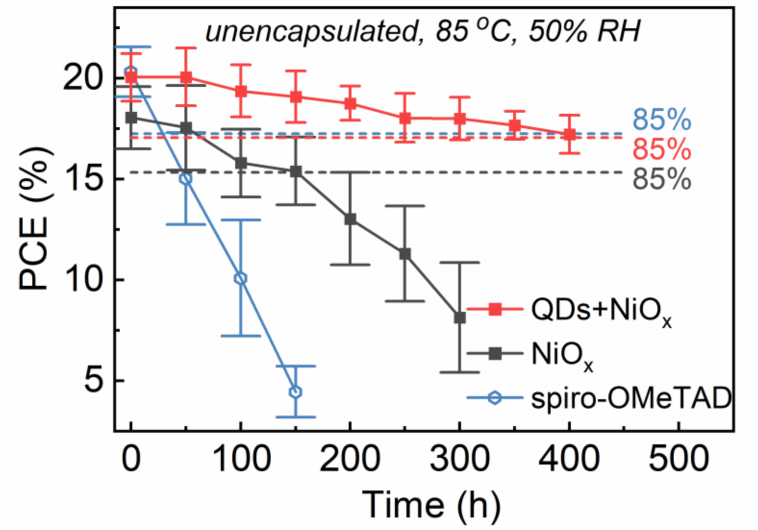

Figure 23. Storage stabilities of unencapsulated devices with NiO_x, QDs-promoted NiO_x and spiro-OMeTAD HTL aged at 85 ^{o}C and 50% RH in air.

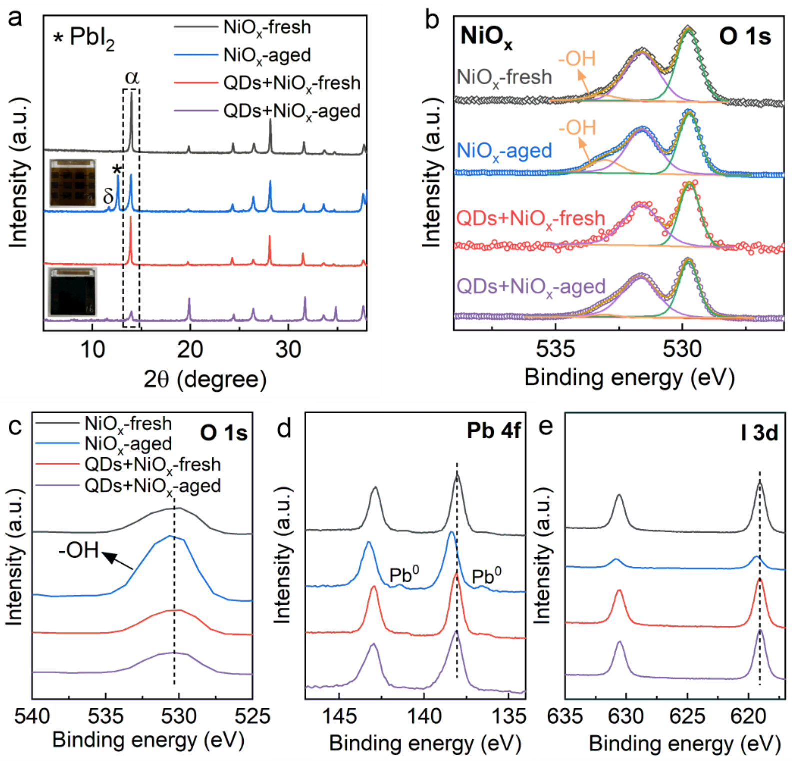

Figure S24. Characterization of NiO_x and perovskite thin films in unencapsulated NiO_x-based devices before and after 200-hour aging at 85 °C in ambient air with 40-50% RH. (a) XRD patterns of fresh and aged NiO_x-devices without or with QD intercalation. (b) XPS of O 1s of NiO_x films in corresponding devices after Au electrode was peeled off. XPS spectra of (c) O 1s, (d) Pb 4f and (e) I 3d of perovskite films in corresponding devices after peeling-off of Au and washing-off of NiO_x.

As shown in the above graphs, XRD data indicated deterioration of α phase and emergence of δ phase for aged perovskite films in bare NiO_x-device, while the perovskite in QDs-promoted device was much more stable with neat α phase after aging. The mechanism for perovskite phase deterioration was further explored by XPS. The amount of hydroxide group in pristine NiO_x film, which mainly resulted from incomplete binding of oleophilic ligands, increased after 85 °C aging in ambient air. The -OH group can bind to Pb^{2+} and thus cause degradation of perovskite. In XPS for perovskite films in aged devices, shift of binding energy of Pb and I also implied PbI_2 generation and Pb^0 appearance. It can be concluded from the above results that one of the reasons for improved stability of QDs-promoted NiO_x-based device is less hydroxide group with better coverage of ligands.

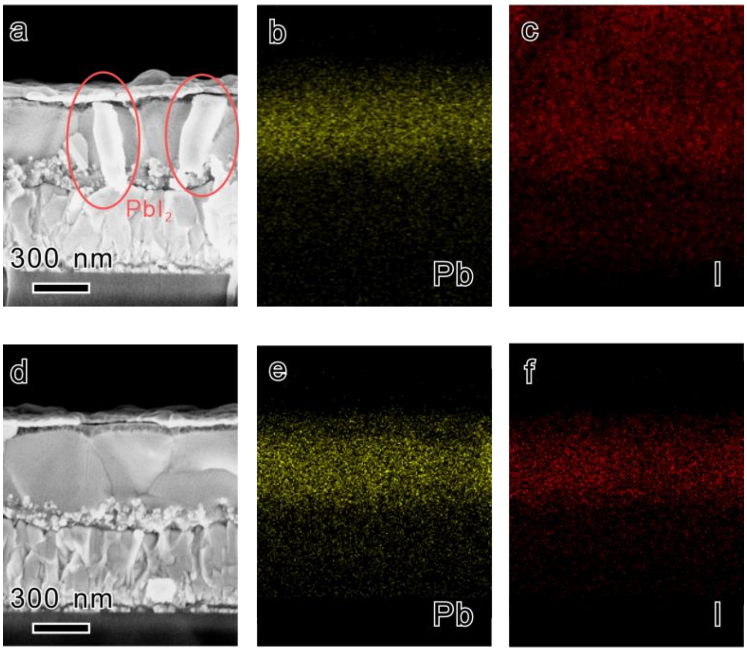

Figure S25. Cross-sectional SEM images and EDX-mappings of 200-hour aged devices (unencapsulated, 85 ^{o}C, 40-50% RH): (a-c) without QD intercalation, (d-f) with QD intercalation. SEM image of the aged device with pristine NiO_x showed the collapse of perovskite crystals and formation of flake-like PbI_2, while the one with QDs remained stable. And EDX mapping of I element indicated the unwanted upward migration of I^- in pristine NiO_x-device, resulting in poor stability. While the QDs-promoted device exhibited negligible I diffusion, indicating that QD intercalation can suppress ion migration.

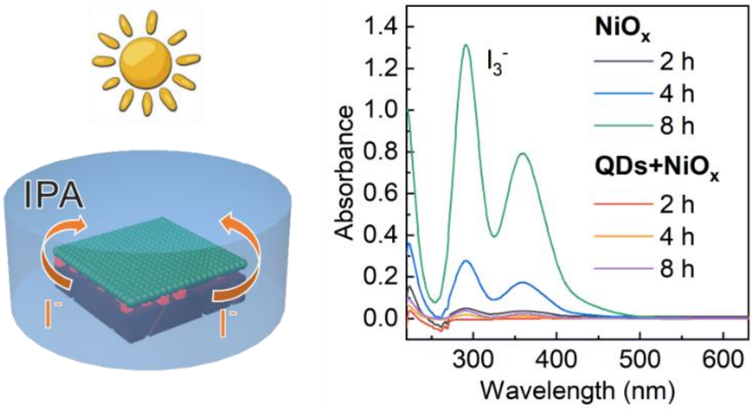

Figure S26. (left) Illustration of aging test by dipping perovskite films covered with NiO_x without or with QD intercalation into IPA under 1-sun illumination. (right) UV-vis absorption spectra of IPA solvent during the aging process. Faster extraction of I_3^- indicated that I^- in pristine perovskite was faced with immigration under illumination.[2] With QD intercalation, I^- was more inert, resulting in stable power output of the NiO_x-device during working condition.

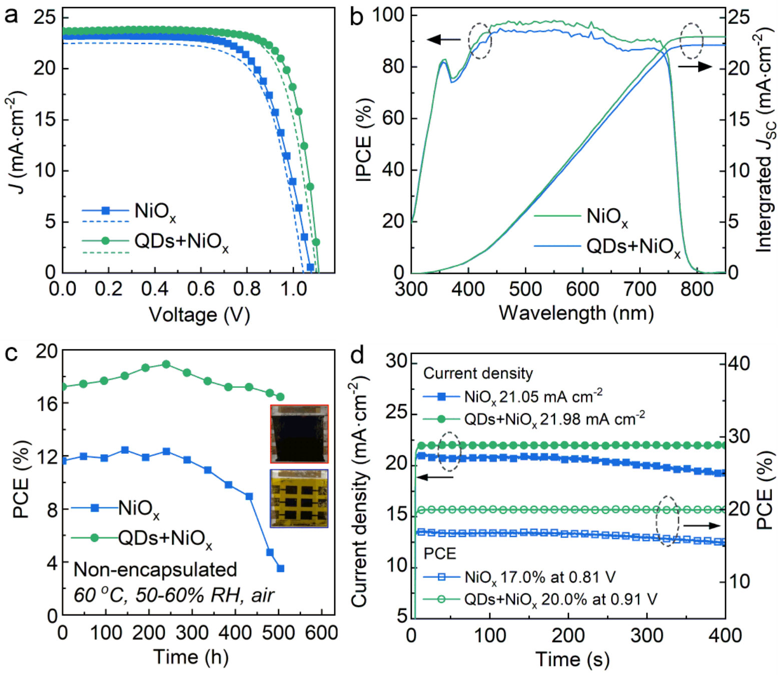

Figure S27. Performance of NiO_x-devices using $MAPbI_3$ without or with QD intercalation. (a) *J*-*V* curves of NiO_x-devices. (b) IPCE curves and corresponding integrated J_{SC} of NiO_x-devices. The integrated J_{SC} of the devices were 22.29 mA·cm^{-2} without QDs and 23.11 mA·cm^{-2} with QD intercalation, respectively. (c) PCE stability of non-encapsulated NiO_x-devices under 85-^{o}C heating and 50-60% RH in air. (d) Output stability of NiO_x-devices measured under 1-sun illumination at maximum power point.

These above results demonstrated that QD intercalation was also useful for the NiO_x-device with pure $MAPbI_3$ films, showing evident enhancements in device's efficiency and stability (Table S3).

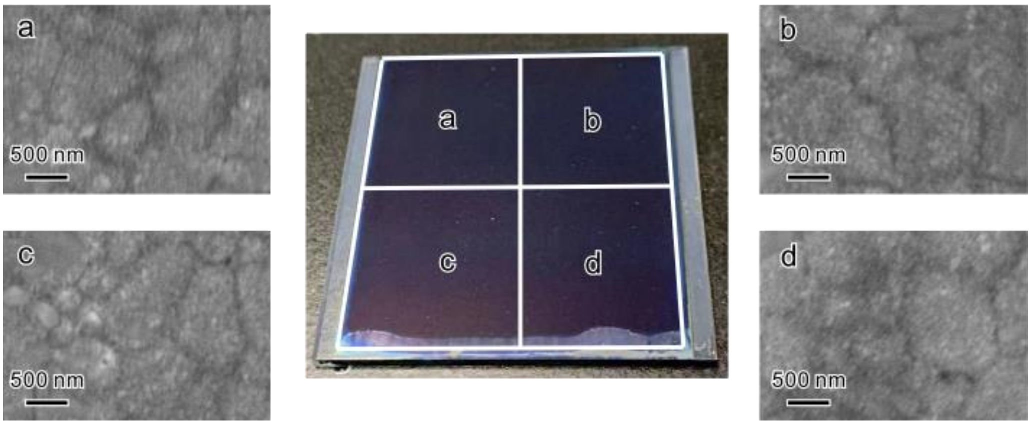

Figure S28. Morphologies of different positions for a 36-cm^2 QDs-promoted NiO_x thin film on perovskite.

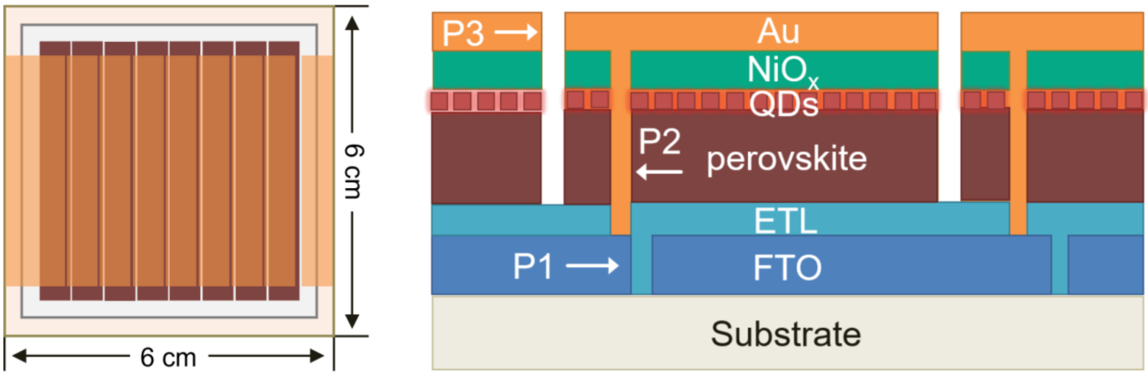

Figure S29. Schematic diagram of plan view and part of cross-sectional view for the NiO_x-based modules with n-i-p structure. The area of FTO substrate was 36 cm^2 and the active area of the module was 18 cm^2. Each module consisted of 8 separate sub-cells.

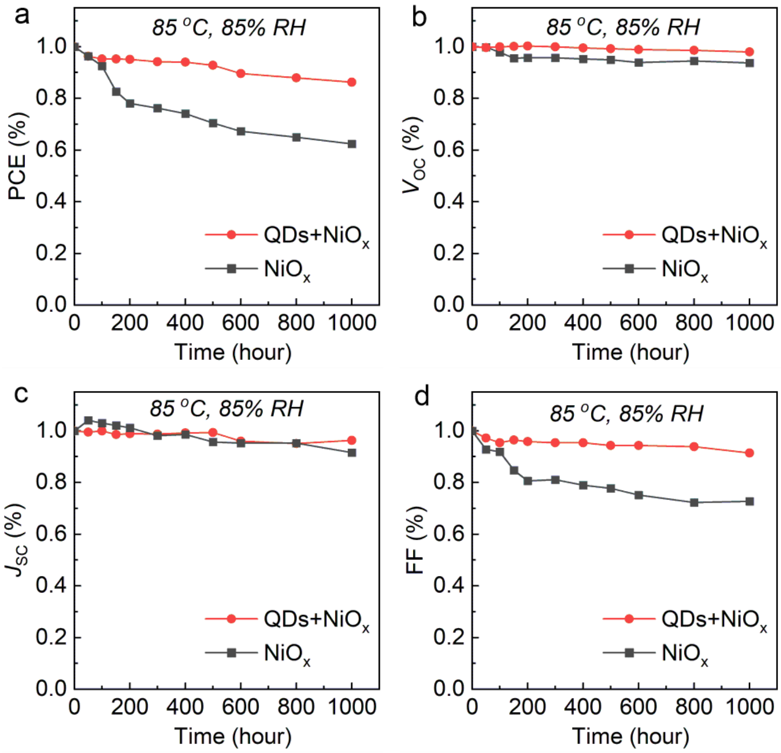

Figure S30. Tracking of (a) PCE, (b) V_{OC}, (c) J_{SC} and (d) FF of encapsulated modules under 85 ^{o}C/85 RH aging condition.

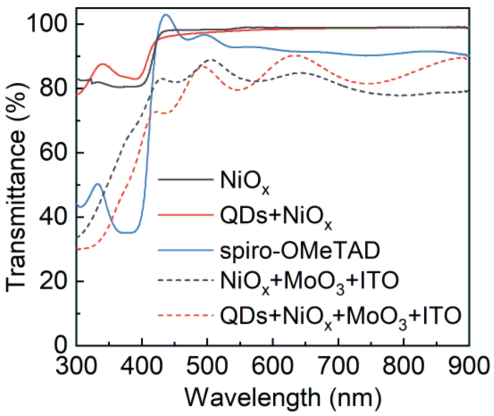

Films	NiO_x	QDs+NiO_x	spiro	NiO_x+MoO_3 +ITO	NiO_x+MoO_3 +ITO
AVT	95.3%	95.6%	83.5%	76.6%	76.2%

Figure S31. Transmittance of NiO_x and QDs-promoted NiO_x thin films without or with top MoO_3/ITO electrode.

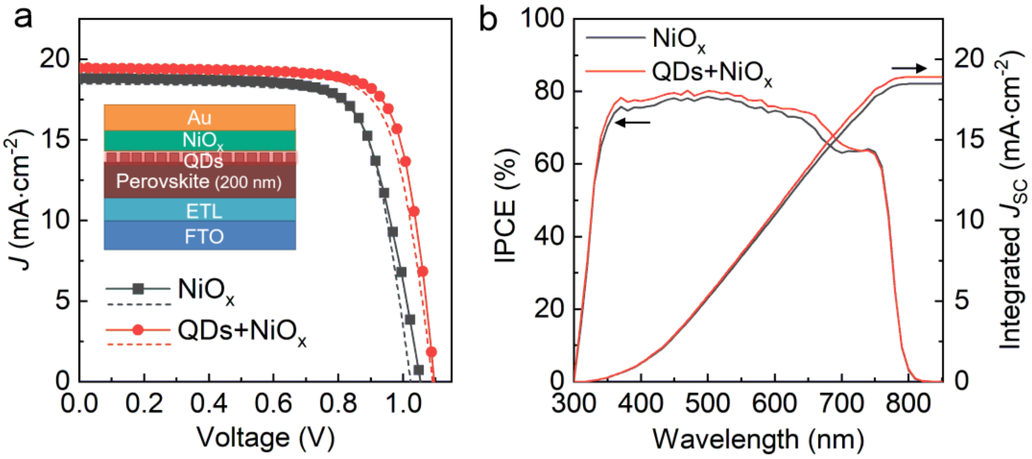

Figure S32. (a) *J-V* scans of NiO_x-PSCs with ultra-thin $FA_{0.85}MA_{0.15}Pb(I_{0.85}Br_{0.15})_3$ film and Au electrode. (b) IPCE curves and integrated J_{SC} of the corresponding devices. The device with QD-NiO_x exhibited high V_{OC} and FF (Table S6), which indicated that defect-less thin perovskite layers were ready for usage in ST-devices.

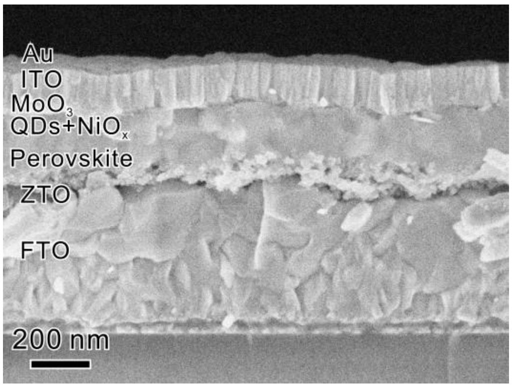

Figure S33. Cross-sectional SEM image of the whole ST-device.

Table S1. Photovoltaic parameters of NiO_x-PSCs with different concentration of NiO_x-NP solutions.

Entry	J_{sc} [mA·cm^{-2}]	V_{oc} [V]	FF [%]	PCE [%]
NiO_x-10 mg/mL	22.79	1.073	73.83	18.05
NiO_x-15 mg/mL	23.62	1.083	73.32	18.75
NiO_x-20 mg/mL	23.66	1.046	71.43	17.68
NiO_x-25 mg/mL	23.82	1.052	68.24	17.10

Table S2. Photovoltaic parameters of NiO_x-PSCs without or with QDs intercalation with perovskite component of $FA_{0.85}MA_{0.15}Pb(I_{0.85}Br_{0.15})_3$ or $MAPbI_3$.

Entry	J_{sc} [mA·cm^{-2}]	V_{oc} [V]	FF [%]	Reverse scan [%]	Forward scan [%]
$FAMAPb(IBr)_3$+NiO_x	23.62	1.083	73.32	18.75	17.51
$FAMAPb(IBr)_3$+QDs+NiO_x	24.45	1.114	79.29	21.59	20.22
$MAPbI_3$+NiO_x	23.24	1.078	67.94	17.02	16.20
$MAPbI_3$+QDs+NiO_x	23.70	1.110	76.11	20.02	19.29

Table S3. Photovoltaic parameters of reported PSCs with NiO_x HTL deposited from NiO_x nanoparticles.

n-i-p structure

Device structure	Solvent	V_{OC} [V]	J_{SC} [mA·cm^{-2}]	FF [%]	PCE [%]	Year	Ref.
FTO/TiO_2/$MAPbI_3$–NiO/Au	DMF	0.82	26.41	56.1	12.14	2016	[3]
FTO/TiO_2/$MAPbI_3$/NiO_x/Au<br>ITO/NiO_x/$MAPbI_3$/PCBM/BCP/Ag	CB	0.88<br>1.03	19.49<br>20.66	53.1<br>74.2	9.11<br>15.90	2017	[4]
FTO/TiO_2/FAMAPb$(IBr)_3$/NiO_x/Au	1-butanol	0.85±0.02	19.04±0.05	57.4±3.2	9.35±0.75	2017	[5]
FTO/TiO_2/$MAPbI_3$/NiO_x/MWCNT	CB	0.91	22.84	76.0	15.80	2017	[6]
FTO/TiO_2/$MAPbI_3$/NiO_x/Au	IPA	0.97	18.12	62.0	10.89	2018	[7]
FTO/SnO_2/CsFAMAPb$(IBr)_3$/NiO_x/Au	CB	1.04	22.53	54.4	12.71	2019	[8]
FTO/TiO_2/CsFAMAPb$(IBr)_3$/NiO_x/Au	CB	0.99	21.90	60.0	13.10	2019	[9]
FTO/TiO_2/CsFAPb$(IBr)_3$/NiO_x/CNT/carbon	CB	0.97	18.30	64.0	11.36	2021	[10]
FTO/TiO_2/ FAMAPb$(IBr)_3$/QDs/NiO_x/Au	CB/CF	1.11	24.45	79.3	21.59		This work

p-i-n structure

Device structure	Solvent	V_{OC} [V]	J_{SC} [mA·cm^{-2}]	FF [%]	PCE [%]	Year	Ref.
FTO/NiO/$MAPbI_3$/PCBM/Ag	ethanol	1.09	17.93	73.8	14.42	2015	[11]
ITO/NiO/$MAPbI_3$/PCBM/Al	ethanol	1.06	19.85	73.0	15.35	2016	[12]
ITO/NiO/$MAPbI_3$/PCBM/LiF/Al	tetradecane	1.04	20.20	74.0	15.40	2016	[13]
glass-ITO/NiO/$MAPbI_3$/PCBM/Ag<br>PEN-ITO/NiO/$MAPbI_3$/PCBM/Ag	DI water	1.07<br>1.04	20.58<br>18.74	74.8<br>68.9	16.47<br>13.43	2016	[14]
ITO/NiO/$MAPbI_3$/PCBM/Ag	ethanol	1.09	19.90	76.9	16.68	2016	[15]
glass-ITO/NiO_x/$MAPbI_3$/C60/Ag<br>PET-ITO/NiO_x/$MAPbI_3$/C60/Ag	DI water	1.03<br>1.00	21.80<br>20.66	78.4<br>70.5	17.60<br>14.53	2016	[16]

ITO/NiO_x/$MAPbI_3$/PCBM/PDINO/Ag	methanol	1.11	20.57	77.0	17.50	2016	[17]
FTO/NiO_x/FAMAPb$(IBr)_3$/PCBM/Au	DI water	1.07	21.21	71.0	16.08	2017	[18]
ITO/NiO_x/$MAPbI_3$/PCBM/Ag	DI water	1.04	21.22	75.0	16.55	2017	[19]
ITO/NiO_x/$MAPbI_3$/PCBM/C_{60}/Ag	DI water	1.11	21.70	76.3	18.40	2017	[20]
FTO/Cu:NiO_x/$MAPbI_3$/$PC_{61}BM$/bis-C_{60}/Ag	ethanol	1.11	21.66	82.3	19.79	2017	[21]
ITO/NiO_x/$MAPbI_3$/PCBM/BCP/Au	toluene	1.06	19.41	75.0	15.47	2018	[22]
ITO/NiO_x/$MAPbI_3$/PCBM/TiNiO_x/Ag	ethanol	1.07	21.88	79.0	18.49	2018	[23]
ITO/NiO_x/$MAPbI_3$/PCBM/TiNiO_x/Ag	water	1.06	21.13	76.0	17.02		
g-ITO/NiO_x/CsFAMAPb$(IBr)_3$/PCBM/ZnO/Al	DI water	1.02	22.20	82.0	18.60	2018	[24]
P-ITO/NiO_x/CsFAMAPb$(IBr)_3$/PCBM/ZnO/Al		1.03	21.20	76.0	16.60		
g-ITO/Cu:NiO_x/$MAPbI_3$/C_{60}/BCP/Ag	water/IPA	1.12	22.28	81.2	20.26	2018	[25]
P-ITO/Cu:NiO_x/$MAPbI_3$/C_{60}/BCP/Ag		1.10	21.45	73.8	17.41		
ITO/Co:NiO_x/$MAPbI_3$/PCBM/BCP/Au	DI water	1.06	17.30	79.0	14.50	2019	[26]
ITO/NiO_x/CsMAFAPb$(IBr)_3$/PCBM/BCP/Ag	DI water	1.14	23.44	82.8	22.13	2020	[27]
ITO/NiO_x/CsMAFAPb$(IBr)_3$/PCBM/BCP/Cr/Au	DI water	1.13	23.24	84.1	22.11	2021	[28]
ITO/NiO_x/CsFAPb$(IBr)_3$/PCBM/C60/BCP/Ag	DMF	1.07	22.10	76.0	17.90	2021	[29]
FTO/NiO_x/$MAPbI_3$/PCBM/Ag	DI water	1.08	21.47	81.3	18.85	2021	[30]

DMF: dimethylformamide; CB: chlorobenzene; IPA: isopropanol; DI water: deionized water;
g-ITO: glass-ITO; P-ITO: PEN-ITO.

Table S4. Photovoltaic parameters of 18-cm^2 solar modules with NiO_x-based HTLs.

Entry	J_{sc} [mA·cm^{-2}]	V_{oc} [V]	FF [%]	Reverse scan [%]	Forward scan [%]
NiO_x	2.88	8.291	70.95	16.94	13.78
QDs+NiO_x	2.98	8.590	74.61	19.10	17.65

Table S5. Photovoltaic parameters of NiO_x-PSCs with ultra-thin $FA_{0.85}MA_{0.15}Pb(I_{0.85}Br_{0.15})_3$ and Au electrode.

Entry	J_{sc} [mA·cm^{-2}]	V_{oc} [V]	FF [%]	Reverse scan [%]	Forward scan [%]
NiO_x+Au	18.85	1.053	72.41	14.37	14.25
QDs+NiO_x+Au	19.45	1.095	77.03	16.40	15.60

Table S6. Photovoltaic parameters of semi-transparent devices with NiO_x-based HTLs.

Entry	J_{sc} [mA·cm^{-2}]	V_{oc} [V]	FF [%]	Reverse scan [%]	Forward scan [%]
NiO_x (FTO side)	18.64	1.017	65.44	12.40	10.82
NiO_x (ITO side)	15.37	0.984	64.96	9.82	7.25
QDs+NiO_x (FTO side)	19.18	1.065	69.79	14.25	12.78
QDs+NiO_x (ITO side)	15.85	1.007	68.68	10.96	9.46

Table S7. Integrated and measured J_{SC} of semi-transparent devices with NiO_x-based HTLs.

Entry	Integrated J_{SC} [mA·cm^{-2}]	Measured J_{SC} [mA·cm^{-2}]	Deviation [%]
NiO_x (FTO side)	18.64	17.93	3.8
NiO_x (ITO side)	15.37	14.75	4.0
QDs+NiO_x (FTO side)	19.18	18.56	3.2
QDs+NiO_x (ITO side)	15.85	15.24	3.8

References:

[1] J. Li, L. Xu, T. Wang, J. Song, J. Chen, J. Xue, Y. Dong, B. Cai, Q. Shan, B. Han, H. Zeng, *Adv. Mater.* **2017**, *29*, 1603885.

[2] W. S. Yang, B. W. Park, E. H. Jung, N. J. Jeon, Y. C. Kim, D. U. Lee, S. S. Shin, J. Seo, E. K. Kim, J. H. Noh, S. I. Seok, *Science* **2017**, *356*, 1376-1379.

[3] Y. S. Wang, W. Y. Rho, H. Y. Yang, T. Mahmoudi, S. Seo, D. H. Lee, Y. B. Hahn, *Nano Energy* **2016**, *27*, 535-544.

[4] Z. Liu, A. Zhu, F. Cai, L. Tao, Y. Zhou, Z. Zhao, Q. Chen, Y. B. Cheng, H. Zhou, *J. Mater. Chem. A* **2017**, *5*, 6597-6605.

[5] J. Cao, H. Yu, S. Zhou, M. Qin, T. K. Lau, X. Lu, N. Zhao, C. P. Wong, *J. Mater. Chem. A* **2017**, *5*, 11071-11077.

[6] Y. L. Yang, H. N. Chen, X. L. Zheng, X. Y. Meng, T. Zhang, C. Hu, Y. Bai, S. Xiao, S. H. Yang, *Nano Energy* **2017**, *42*, 322-333.

[7] K. C. Icli, M. Ozenbas, *Electrochim. Acta* **2018**, *263*, 338-345.

[8] J. Tirado, M. Vásquez-Montoya, C. Roldán-Carmona, M. Ralaiarisoa, N. Koch, M. K. Nazeeruddin, F. Jaramillo, *ACS Appl. Energy Mater.* **2019**, *2*, 4890-4899.
[9] R. Kaneko, H. Kanda, K. Sugawa, J. Otsuki, A. Islam, M. K. Nazeeruddin, *Sol. RRL* **2019**, *3*, 1900172.
[10] P. Kartikay, D. Sadhukhan, A. Yella, S. Mallick, *Sol. Energy Mater Sol. Cells* **2021**, *230*, 111241.
[11] X. Yin, M. Que, Y. Xing, W. Que, *J. Mater. Chem. A* **2015**, *3*, 24495-24503.
[12] S. S. Mali, H. Kim, S. E. Shim, C. K. Hong, *Nanoscale* **2016**, *8*, 19189-19194.
[13] U. Kwon, B. G. Kim, D. C. Nguyen, J. H. Park, N. Y. Ha, S. J. Kim, S. H. Ko, S. Lee, D. Lee, H. J. Park, *Sci. Rep.* **2016**, *6*, 30759.
[14] X. Yin, P. Chen, M. Que, Y. Xing, W. Que, C. Niu, J. Shao, *ACS Nano* **2016**, *10*, 3630-3636.
[15] X. Yin, J. Liu, J. Ma, C. Zhang, P. Chen, M. Que, Y. Yang, W. Que, C. Niu, J. Shao, *J. Power Sources* **2016**, *329*, 398-405.
[16] H. Zhang, J. Cheng, F. Lin, H. He, J. Mao, K. S. Wong, A. K. Jen, W. C. Choy, *ACS Nano* **2016**, *10*, 1503-1511.
[17] Y. Hou, W. Chen, D. Baran, T. Stubhan, N. A. Luechinger, B. Hartmeier, M. Richter, J. Min, S. Chen, C. O. Quiroz, N. Li, H. Zhang, T. Heumueller, G. J. Matt, A. Osvet, K. Forberich, Z. G. Zhang, Y. Li, B. Winter, P. Schweizer, E. Spiecker, C. J. Brabec, *Adv. Mater.* **2016**, *28*, 5112-5120.
[18] K. Lee, J. Ryu, H. Yu, J. Yun, J. Lee, J. Jang, *Nanoscale* **2017**, *9*, 16249-16255.
[19] J. Ciro, D. Ramirez, M. A. Mejia Escobar, J. F. Montoya, S. Mesa, R. Betancur, F. Jaramillo, *ACS Appl. Mater. Interfaces* **2017**, *9*, 12348-12354.
[20] Q. Wang, C. C. Chueh, T. Zhao, J. Cheng, M. Eslamian, W. C. H. Choy, A. K. Jen, *ChemSusChem* **2017**, *10*, 3794-3803.
[21] K. Yao, F. Li, Q. He, X. Wang, Y. Jiang, H. Huang, A. K. Y. Jen, *Nano Energy* **2017**, *40*, 155-162.
[22] J. Tang, D. Jiao, L. Zhang, X. Z. Zhang, X. X. Xu, C. Yao, J. H. Wu, Z. Lan, *Sol Energy* **2018**, *161*, 100-108.
[23] J. He, E. Bi, W. Tang, Y. Wang, Z. Zhou, X. Yang, H. Chen, L. Han, *Sol. RRL* **2018**, *2*, 1800004.
[24] M. Najafi, F. Di Giacomo, D. Zhang, S. Shanmugam, A. Senes, W. Verhees, A. Hadipour, Y. Galagan, T. Aernouts, S. Veenstra, R. Andriessen, *Small* **2018**, *14*, e1702775.
[25] W. Chen, Y. H. Wu, J. Fan, A. B. Djurisic, F. Z. Liu, H. W. Tam, A. Ng, C. Surya, W. K. Chan, D. Wang, Z. B. He, *Adv. Energy Mater.* **2018**, *8*, 1703519.
[26] R. Kaneko, T. H. Chowdhury, G. Wu, M. E. Kayesh, S. Kazaoui, K. Sugawa, J. J. Lee, T. Noda, A. Islam, J. Otsuki, *Sol Energy* **2019**, *181*, 243-250.
[27] P. Ru, E. Bi, Y. Zhang, Y. Wang, W. Kong, Y. Sha, W. Tang, P. Zhang, Y. Wu, W. Chen, X. Yang, H. Chen, L. Han, *Adv. Energy Mater.* **2020**, *10*, 1903487.
[28] Q. Cao, Y. Li, H. Zhang, J. Yang, J. Han, T. Xu, S. Wang, Z. Wang, B. Gao, J. Zhao, X. Li, X. Ma, S. M. Zakeeruddin, W. E. I. Sha, X. Li, M. Grätzel, *Sci. Adv.* **2021**, *7*, eabg0633.
[29] M. Michalska, M. A. Surmiak, F. Maasoumi, D. C. Senevirathna, P. Chantler, H. Li, B. Li, T. Zhang, X. Lin, H. Deng, N. Chandrasekaran, T. A. N. Peiris, K. J. Rietwyk, A. S. R. Chesman, T. Alan, D. Vak, U. Bach, J. J. Jasieniak, *Sol. RRL* **2021**, *5*, 2100342.
[30] P. H. Lee, T. T. Wu, C. F. Li, D. Głowienka, Y. H. Sun, Y. T. Lin, H. W. Yen, C. G. Huang, Y. Galagan, Y. C. Huang, W. F. Su, *Chem. Eng. J.* **2021**, *412*, 128746.
